# Climatic Conditions and Amine Loading Impact the Performance of Laminate-Supported Poly(ethylenimine) Direct Air Capture Sorbents

**DOI:** 10.1021/jacsau.5c01294

**Published:** 2025-11-25

**Authors:** UnJin Ryu, Youn Ji Min, Wenyang Zhao, Yunseok Lee, Matthew J. Realff, Christopher W. Jones

**Affiliations:** School of Chemical & Biomolecular Engineering, 1372 Georgia Institute of Technology, 311 Ferst Dr, Atlanta, Georgia 30332, United States

**Keywords:** direct air capture, CO_2_ capture, polyethylenimine, subambient, humidity, gas–solid contactor

## Abstract

Direct air capture (DAC) of CO_2_ is a critical technology to combat climate change, offering a scalable approach to mitigate atmospheric CO_2_ accumulation. Despite advancements in adsorbent materials, the performance of DAC systems remains highly sensitive to environmental conditions, such as temperature and humidity. Poly­(ethylenimine) (PEI)-functionalized silica has emerged as a promising sorbent due to its high CO_2_ capacity and tunable properties. However, optimizing its performance across diverse climatic conditions requires a deeper understanding of how the PEI loading impacts the adsorption capacity and kinetics under varying temperatures and humidities. This study reports the CO_2_ and H_2_O adsorption kinetics and capacities, desorption behavior, and speciation of adsorbed CO_2_ using silica/PEI adsorbents supported within expanded poly­(tetrafluoroethylene) (ePTFE) sheets across a wide range of temperatures and humidities, representing the diversity of climates found across the United States. With the obtained data, guidelines for selecting and optimizing PEI-loaded silica-based sorbents for DAC applications are developed. These findings provide a framework for tailoring sorbents to specific climatic scenarios, ensuring a reliable and efficient DAC performance.

## Graphical abstract

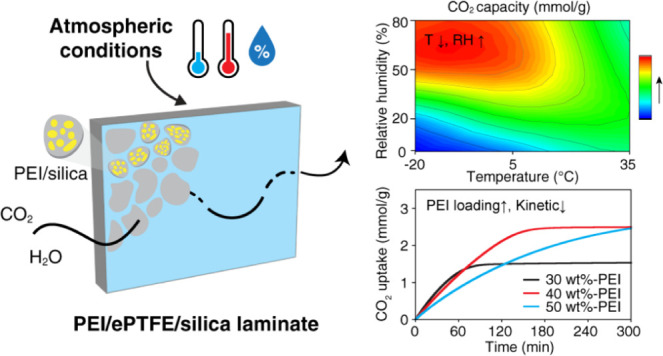

## Introduction

Direct air capture (DAC) is a promising negative emission technology that captures carbon dioxide directly from the atmosphere, offering flexible deployment regardless of geographic location or proximity to industrial emissions sources. Since its early conceptual development in the early 2000s, DAC technology has progressed to the stage of commercial demonstration with several pilot-scale systems currently in operation worldwide by companies such as Climeworks, Global Thermostat and others.^1,2^

Various materials have been explored for DAC, including alkali carbonates and hydroxides,^3,4^ metal–organic frameworks (MOFs),^5^ zeolites,^6^ ionic liquids,^7^ and amine-modified materials.^8–10^ Among these, amine-functionalized porous solids have attracted particular attention due to their CO_2_ selectivity and ability to chemically bind CO_2_ even at the low partial pressures found in ambient air.^10^ These composite materials typically consist of a porous support such as mesoporous silica, alumina, polymers, or metal–organic frameworks (MOFs) impregnated with amine species. Amine polymers, oligomers, and small molecules have been studied according to their linear or branched structures and type(s) of amines, such as poly­(ethylenimine) (PEI), tetraethylenepentamine (TEPA), or mono/diethanolamine (MEA/DEA).^12^ Extensive research has been undertaken to understand the interaction(s) between the aminopolymers and the support pore structure, and it has been shown that the pore size and surface chemistry significantly affect the CO_2_ sorption capacity, mechanism, and kinetics.^10,13–16^ However, aminopolymer-based sorbents can undergo structural and chemical changes under harsh regeneration or humid conditions, including thermal, CO_2_-induced, O_2_-induced, and hydrothermal degradation pathways that reduce their long-term stability.^17,18^

While progress in designing sorbents with enhanced CO_2_ selectivity has been substantial, an equally critical challenge in DAC arises from the need to process immense volumes of air to capture meaningful quantities of CO_2_, given its dilute atmospheric concentration (∼0.04 vol %). Conventional sorbent forms, often developed as loose powders and tested in fixed-bed configurations, suffer from poor heat and mass transfer efficiency and a significant pressure drop, limiting their scalability. To overcome these transport and engineering constraints, structured gas–solid contactors with high mechanical robustness have emerged as a promising platform for deploying solid-supported amine sorbents in large-scale DAC systems,^19–21^ including polymeric organic–inorganic composites formulated by extrusion,^22^ fiber spinning,^23,24^ lamination,^25^ and 3D printing.^26,27^

In practical DAC applications, the performance of amine-functionalized sorbents can be affected by environmental conditions, especially temperature and relative humidity (RH), which vary significantly depending on the location and season.^28^ It has been demonstrated that under humid conditions, CO_2_ can react not only with primary and secondary amines but also (to a limited extent) with tertiary amines via bicarbonate formation.^29–31^ In addition to bicarbonate, other species such as carbamic acid, ammonium carbamate ion-pairs, and zwitterionic intermediates have been also observed, and these are supported by in situ IR and NMR spectroscopy.^32–37^ These results highlight the important role of water in modifying the CO_2_ adsorption mechanism. However, previous studies have focused on a narrow range of temperature and RH conditions, and there remains a lack of systematic understanding of how a broad range of environmental parameters interact to affect the sorbent performance.

In this study, we investigate PEI-impregnated ePTFE/silica sorbents under simulated atmospheric conditions to evaluate their CO_2_ capture behavior over a wide range of temperatures (−20 to 35 °C) and RH (0–80%). Commercial mesoporous silica was selected as the support material due to its high surface area, large pore volume, and structural stability during the DAC process. Branched poly­(ethylenimine) (PEI, MW 800) was used as the functional component, which contains a mixture of primary, secondary, and tertiary amines that enable various CO_2_ adsorption pathways. The PEI/silica composite was incorporated into a mechanically robust and stackable ePTFE-based laminate sheet for easy integration into fixed-bed DAC systems.^25^ The CO_2_ capacity, adsorption kinetics, moisture absorption behavior, and regeneration characteristics were evaluated under these simulated atmospheric conditions to derive optimal adsorbent compositions for different operational windows.

## Results and Discussion

### Characterization of PEI-Impregnated ePTFE/Silica Sorbents

The ePTFE/silica composite material, a self-standing sheet with a thickness of 0.7 mm,^25^ is illustrated in Figure 1.^25^ This material was chosen as the support for the aminopolymer due to its advantageous properties for adoption in DAC systems. First, the self-standing sheet design enables easy stacking, facilitating the construction of a low-pressure drop contactor system. This stackable structure is also easily managed and maintained, as individual sheets can be conveniently replaced when needed. Additionally, the hydrophobic nature of the ePTFE component offers significant advantages. Hydrophobic components can lower the overall water uptake of the system, reducing the parasitic energy load due to water desorption. This combination of structural stability, ease of maintenance, and moisture resistance makes the ePTFE/silica composite a promising support material for DAC applications and was thus chosen for this study, where we sought to comprehensively characterize a sorbent material across multiple practical operational conditions. These advantages are tempered by potential disadvantages of ePTFE, such as its low thermal conductivity and its fluorinated nature.

**Figure 1:**
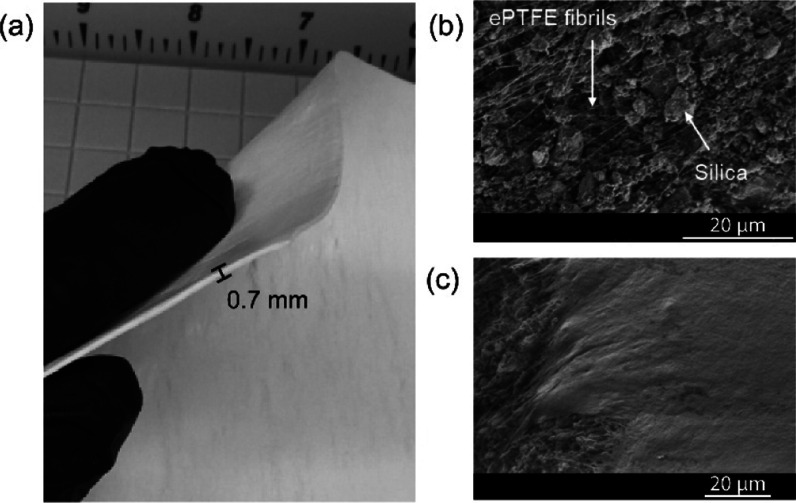
(a) Optical image and (b) cross-sectional and (c) top view SEM images of the self-standing ePTFE/silica substrate.

The PEI-impregnated ePTFE/silica materials (PEI/ePTFE/silica) were prepared by a wet impregnation method developed for structured sorbents developed in our group.^25,38^ This preparation utilized the equilibrium distribution of PEI between a PEI/MeOH solution and an ePTFE/silica substrate. Samples with different PEI loadings were obtained by adjusting the PEI concentration (5–40 wt %) in the PEI/MeOH solution. The PEI loading was quantified by the mass difference before and after amine impregnation of the ePTFE/silica materials and corroborated by pyrolysis thermogravimetric analysis (TGA) (Figure S1 and Table S1). The PEI loading of the sorbent (PEI/ePTFE/silica) increased progressively with the concentration of PEI in the PEI/MeOH solution (Table 1 and Figure 2). The PEI loading ranged from 13.2 wt % for Entry 1 (5 wt % PEI solution) to 49.1 wt % for Entry 8 (40 wt % PEI solution).

**Table 1:** Physical Properties of ePTFE/Silica Materials before and after PEI Impregnation

entry	PEI concentration in PEI/MeOH (wt %)	PEI loading (wt %)	BET surface area (m^2^/g_sorbent_)	BJH pore volume (mL/g)
bare ePTFE/silica			256	1.18
1	5	13.2	179	0.97
2	10	20.5	137	0.85
3	15	27.0	110	0.76
4	20	31.9	102	0.66
5	25	38.3	64	0.43
6	30	47.6	36	0.24
7	35	49.1	23	0.16
8	40	52.9	14	0.09

**Figure 2:**
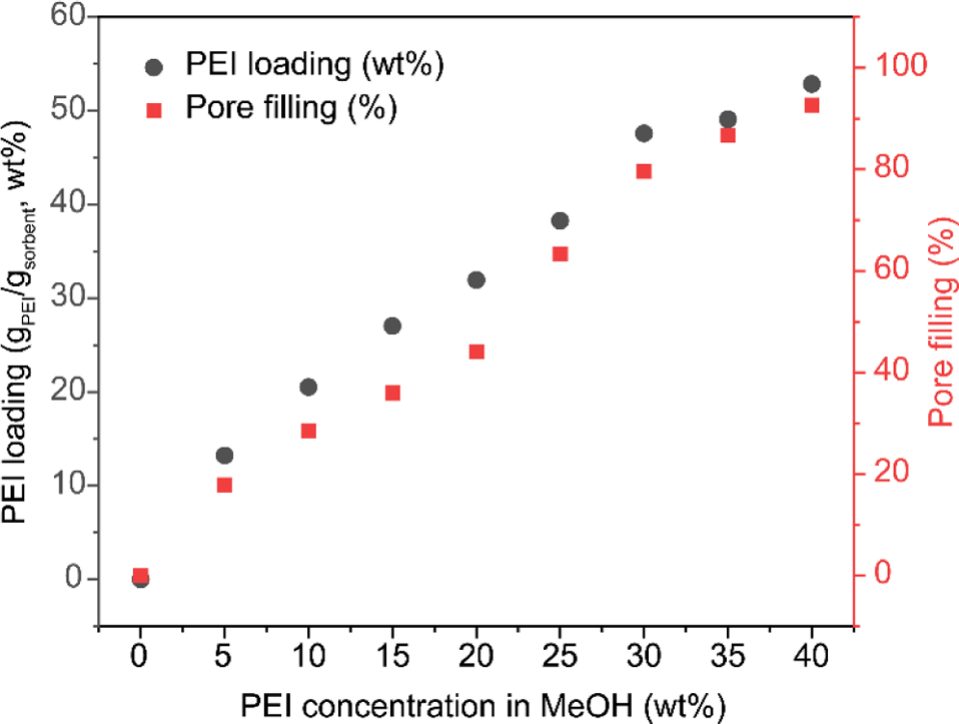
PEI loading and pore filling (%) depending on PEI concentration in a PEI/MeOH impregnation solution.

SEM images suggested that the PEI polymer is uniformly distributed within the ePTFE/silica materials without any noticeable aggregation on the surface or between silica particles (Figure S2). SEM-EDS analysis detected the nitrogen element only in the silica particles, indicating that the hydrophilic PEI polymer preferentially partitioned into the mesoporous silica rather than the hydrophobic ePTFE. These observations are consistent with the results of the N_2_ physisorption isotherms (Figure S3a and Table 1), which showed that the silica pore volume and pore radius decreased with increasing PEI loading (Figure S3b,c). The reduction in the size of the hysteresis loop, which is characteristic of mesoporous silica,^39^ further supports that the PEI polymer is distributed in the silica mesopores and fills the available space while maintaining a relatively uniform loading across the silica particles. To clarify whether the presence of ePTFE affects the overall pore structure of the silica, we compared PEI-impregnated ePTFE/silica materials to PEI-loaded silica powders (PEI/silica). ePTFE provides macroporous networks between silica particles that cannot be accurately measured by this technique. Therefore, the nitrogen isotherms were recalculated based on the combined mass of silica and PEI, excluding ePTFE (Figure S4). As shown in Figure S4c, when normalized to comparable PEI loading levels (g_PEI_/g_silica_), both materials exhibited similar N_2_ adsorption isotherms. Furthermore, the corresponding pore filling values, derived from these isotherms and presented in Figure S4d, were also nearly identical across the two sample types. These results indicate that the presence of ePTFE does not significantly hinder accessibility or pore utilization of the silica support. As a result, pore filling increased proportionally with the amount of PEI loaded, and it was confirmed that PEI/ePTFE/silica samples with various PEI loadings were successfully prepared (Figure 2).

### Dry CO_2_ Adsorption Performance at 35 °C: Baseline Assessment

The CO_2_ adsorption performance of PEI/ePTFE/silica materials was evaluated to determine the optimal PEI loading range for further studies under simulated atmospheric conditions. The eight PEI/ePTFE/silica samples with different PEI loadings were punched into uniform 5 mm diameter rounds to ensure consistency during the evaluation. The adsorption experiments were performed under dry conditions at 35 °C using a thermogravimetric analysis (TGA) system with a gas mixture of 400 ppm of CO_2_ balanced with N_2_ to simulate the ambient atmospheric environment.

The 400 ppm of CO_2_ adsorption capacities of the PEI/ePTFE/silica sorbents, depicted in Figure 3 (black dot), demonstrates a clear trend of increasing capacity with higher PEI loading. The CO_2_ capacity was calculated based on the total mass of PEI-loaded ePTFE/silica sorbents (g_sorbent_), which includes both the ePTFE/silica support and the impregnated PEI. The ePTFE/silica sample with the lowest PEI loading of 13.2 wt % exhibited the lowest adsorption capacity of 0.25 mmol of CO_2_/g_sorbent_. The capacity increased gradually with higher PEI loadings, reaching 1.52 mmol of CO_2_/g_sorbent_ at 38.3 wt % PEI/ePTFE/silica. However, beyond this point, the rate of capacity increase declined sharply. The maximum capacity of 1.74 mmol of CO_2_/g_sorbent_ was achieved with the highest PEI loading of 52.9 wt %. This is because the higher PEI loading increases the number of amine moieties, providing more active sites for CO_2_ adsorption and thereby enhancing the overall capacity. However, the amine efficiency (mmol of CO_2_/mmol of N), which represents the amount of CO_2_-adsorbed per amine site, was reduced at higher loadings (Figure 3, red dots). This is hypothesized to be due to the inability of CO_2_ to reach amine sites because of transport limitations within the material inside the heavily loaded pores. In this study, the amine sites in the branched PEI (MW 800, Aldrich) used for amine efficienct calculations include primary, secondary, and tertiary amines. The total number of nitrogen species was calculated based on a previous study.^40^

**Figure 3:**
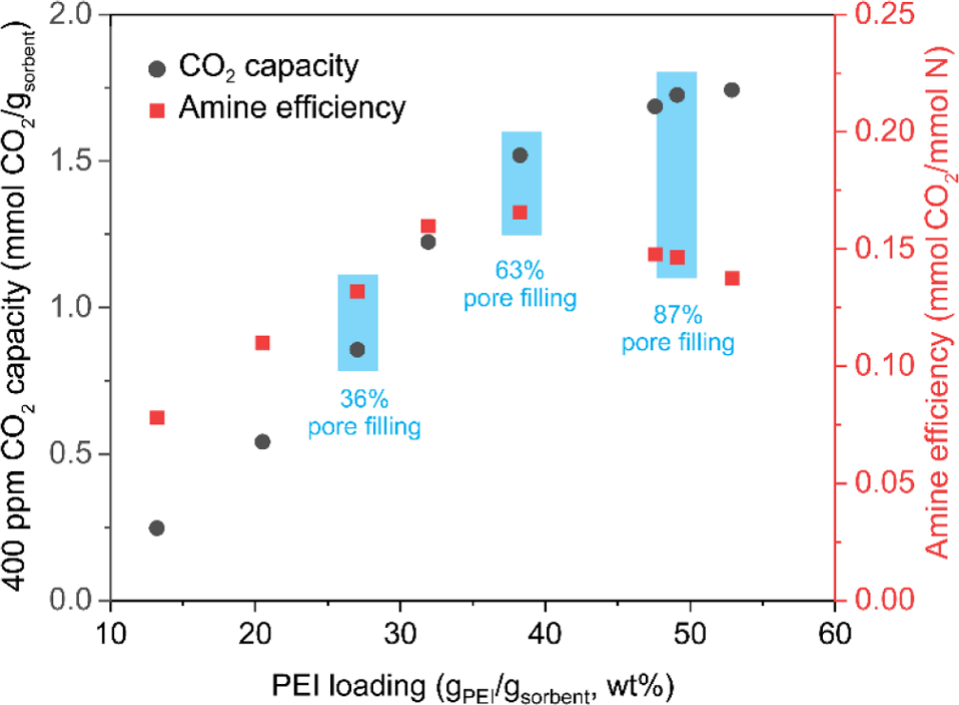
CO_2_ adsorption capacity at 400 ppm of CO_2_/N_2_ and corresponding amine efficiency of ePTFE/silica sorbents with varying PEI loadings at 35 °C using TGA analysis.

Amine efficiency initially increased with PEI loading, reaching a maximum of 0.17 mmol of CO_2_/mmol of N at 38.3 wt % PEI loading (equivalent to 63% pore filling). However, as noted above, beyond this point, efficiency began to decrease. The initial increase is caused by a decreasing fraction of sites that are involved in interactions with the silica surface. As mentioned above, the decline in capacity after reaching a maximum value may be attributed to the reduced accessibility of amine sites due to increased polymer aggregation and diffusion limitations within the pore structure. As the PEI loading increases, the densely packed aminopolymer chains can obstruct CO_2_ diffusion and lead to a reduction in the effective utilization of amine sites despite the higher total nitrogen content.^41^ This is further supported by the absolute CO_2_ uptake profiles shown in Figure S5, which show that higher PEI loadings took longer to approach the equilibrium or pseudoequilibrium capacity. This slower uptake is due to increased diffusion barriers in adsorbents with a higher PEI content. These barriers arise not only from amine sites buried within the PEI domain but also from the reduced rate of CO_2_ diffusion through the PEI chains as the adsorbent becomes saturated. Adsorption as carbamates leads to amine cross-linking by CO_2_, further slowing the overall CO_2_ uptake rate.^32,42,43^ This cross-linking behavior is further supported by DSC analysis (Figure S6), which shows that the glass transition temperature (*T*_g_) of both bulk PEI and PEI/ePTFE/silica increases after saturation under dry CO_2_ following thermal activation under N_2_. This *T*_g_ increase suggests a reduction of polymer chain mobility from CO_2_-bridged amine cross-linking.^44^ CO_2_ diffusion can vary significantly depending on the humidity and temperature conditions. Temperature has a significant impact on the mobility of PEI chains, and this sensitivity can be either advantageous or disadvantageous depending on the DAC operating temperature, as it affects both the CO_2_ uptake capacity (thermodynamics) and the diffusion rate (kinetics)­within the polymer matrix. Additionally, the presence of water molecules influences the available pore space, the CO_2_ adsorption mechanism, and the mobility of PEI, further impacting performance, as discussed below.

To comprehensively investigate these effects of humidity and temperature, we selected three materials with varying PEI loadings for further evaluation under simulated atmospheric conditions. The first material, containing 40 wt %-PEI (medium pore filling, 63%), was selected due to its maximal amine efficiency under dry conditions at 35 °C. The second material, with 30 wt %-PEI (low pore filling, 36%), exhibits both lower capacity and amine efficiency compared to the 40 wt % PEI/ePTFE/silica. In contrast, the third material, with 50-wt % PEI (high pore filling, 87%), achieves a higher capacity but does so with reduced amine efficiency relative to the 40 wt % PEI/ePTFE/silica.

### Fixed-Bed Contactor under Various Potential DAC Operating Environments

To determine the optimal PEI loading for the CO_2_ sorbents, the adsorption performance was evaluated under simulated atmospheric conditions. The experimental design considered the diverse climate conditions of the United States, which can be categorized into six major climate zones.^45,46^ These zones exhibit an average daily temperature fluctuation of approximately 20 °C, with seasonal variations ranging between 15 and 25 °C. Daily RH differences of up to 20% are also common across these regions (Figure S7 and Table S2).^46,47^ To replicate real-world atmospheric conditions, CO_2_ adsorption experiments were conducted at three temperatures: −20 and 5 °C to represent cold or cool conditions and 35 °C to simulate warm climate conditions. Each temperature was tested under varying RH levels of 0%, 20%, 50%, and 80%. The CO_2_ adsorption performance of three different PEI loading samples (30 wt %-, 40 wt %- and 50 wt %-PEI) was evaluated under these 12 conditions, providing a comprehensive evaluation of these sorbents’ performance across diverse potential DAC operating environments (Figure 4a).

**Figure 4:**
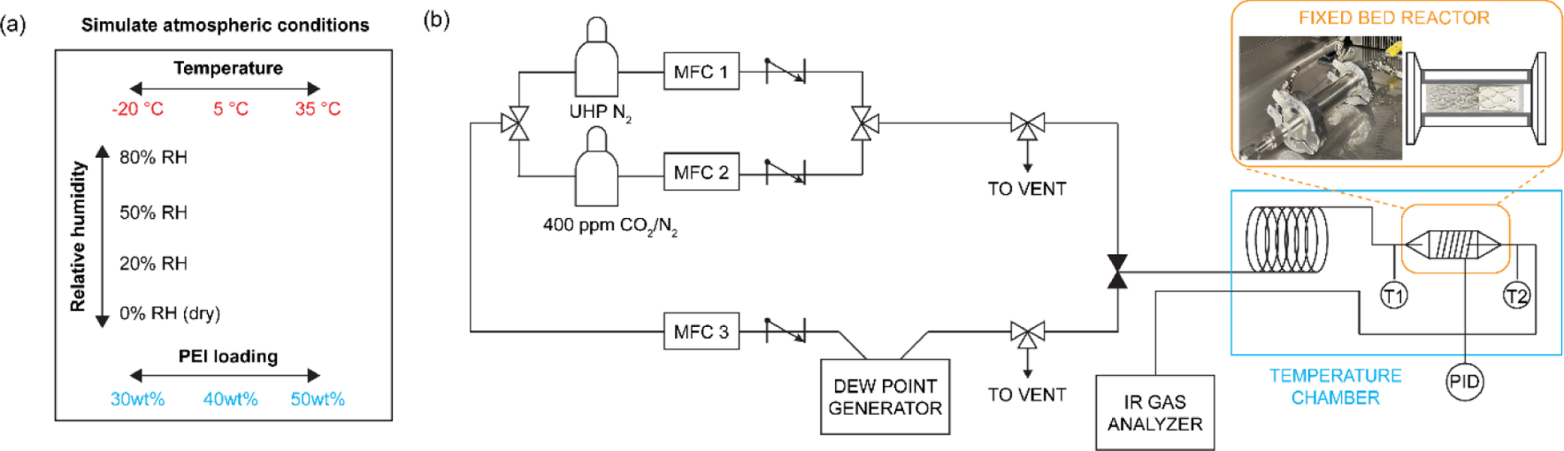
(a) Simulated atmospheric conditions were evaluated under 12 conditions combining three temperatures (−20 °C, 5 °C, and 35 °C) with four RH levels (0%, 20%, 50%, and 80%). (b) P&ID of the custom-built fixed-bed setup. The reactor temperature is controlled by a temperature chamber operating from −20 to 100 °C. Gas flow humidity is regulated using a dew point generator. Subambient humidified gas is generated by mixing dry and humidified gas streams.

The CO_2_ adsorption experiments at 400 ppm of CO_2_/N_2_ flow were conducted using a customized fixed-bed system designed to simulate controlled environmental conditions. As shown in Figure 4b, this system comprises four key components: temperature control, humidity control, a fixed-bed reactor, and a gas detector. For the evaluation, a single sheet of the material was cut into dimensions of 0.5″ × 1″ or 0.5″ × 2″ and positioned vertically in the sample holder (Figure S8). In this study, a presaturation method was adopted to evaluate the adsorption of humid 400 ppm of CO_2_/N_2_ on materials with preadsorbed H_2_O (adsorbed from humid N_2_). This approach reflects real-world scenarios where residual moisture remains in the material after typical DAC regeneration methods, such as steam stripping or moisture-swing regeneration.

### Effect of PEI Loading, Temperature, and Humidity on CO_2_ Adsorption

To comprehensively evaluate the CO_2_ uptake performance of the PEI/ePTFE/silica sorbents, contour maps are provided to illustrate the interdependence of the PEI loading, temperature, and humidity on the CO_2_ capacity. The CO_2_ capacity of each PEI/ePTFE/silica material showed a similar trend under various temperature and humidity conditions, as shown in Figure 5a–c. All materials exhibited the highest CO_2_ capacity (represented in red) at cool to cold temperatures (−20°C < *T* < 5 °C) with 50 – 80% RH, while the lowest CO_2_ capacity (represented in blue) was observed under cold, dry conditions (0% RH) (Table S3).

**Figure 5:**
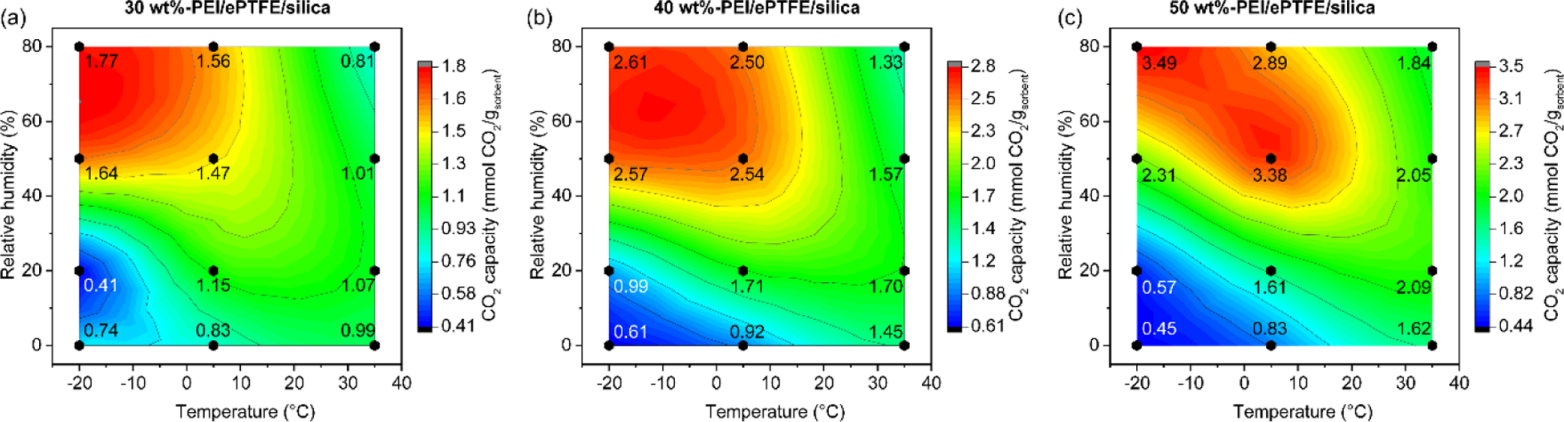
Contour map of CO_2_ capacity at different temperatures and different relative humidities for (a) 30 wt %-PEI/ePTFE/silica, (b) 40 wt %-PEI/ePTFE/silica, and (c) 50 wt %-PEI/ePTFE/silica using a fixed-bed system. The contour levels were set based on the capacity ranges observed for each sample: (a) 0.41–1.8 mmol/g, (b) 0.61–2.8 mmol/g, and (c) 0.44–3.5 mmol/g.

Generally, the CO_2_ adsorption mechanism on aminopolymer/silica sorbents can differ between dry and humid environments. We included eqs 1–6 in this work to represent the possible CO_2_ adsorption mechanisms on amine-based sorbents under dry and humid conditions, as summarized by Panda et al.^48^ Under dry conditions, CO_2_ reacts with primary or secondary amines to initially form a zwitterion intermediate eq 1, which then undergoes proton transfer with another amine (free base) to form a stable ammonium carbamate eq 2. Equation 4 represents a single-step, water-assisted CO_2_ adsorption mechanism, where water acts as a proton acceptor instead of forming a zwitterion intermediate.^49,50^ Additionally, there is a carbamic acid mechanism that involves the formation of carbamic acid as an intermediate (eq 5),^50^ which can subsequently react with another amine to form ammonium carbamate (eq 6).^51,52^ In contrast, under humid conditions, an alternative water-assisted reaction pathway (eq 3) is possible, where water molecules act as proton acceptors to promote the formation of ammonium bicarbonate via a low energy barrier (∼21 kcal mol^–1^).^48^ This reaction mechanism allows even tertiary amines to participate in CO_2_ capture, though typically only at higher CO_2_ pressures. The formation of bicarbonate species is slower than that of ammonium carbamates, and unlike aqueous amines, such species are generally not observed by IR spectroscopy in immobilized/impregnated amine sorbents, though they have been observed at long times and high water loadings by NMR methods.^29,30,33,37^ Carbamates and carbamic acid species dominate the surface of most CO_2_-saturated amine sorbent surfaces under most conditions.

Zwitterion mechanism (type I)

1 $${CO}_{2}+{RNH}_{2}⇄{{RNH}_{2}}^{+}{COO}^{-}\left(E_{a}=∼40-50\;kcal\;{mol}^{-1}\right)$$

2 $${{RNH}_{2}}^{+}{COO}^{-}+{RNH}_{2}⇄RNH{COO}^{-}+{{RNH}_{3}}^{+}\left(E_{a}=∼13.2\;kcal\;{mol}^{-1}\right)\left(dry\;conditions\right)$$

3 $${CO}_{2}+{RNH}_{2}+H_{2}O⇄{{RNH}_{3}}^{+}+H{{CO}_{3}}^{-}\left(E_{a}=∼21\;kcal{\;mol}^{-1}\right)\left(humid\;conditions\right)$$

Single-step mechanism (type II)

4 $${CO}_{2}+{RNH}_{2}\cdot \cdot \cdot B⇄RNH{COO}^{-}\cdot \cdot \cdot {BH}^{+}\left(E_{a}=∼10-26\;kcal{\;mol}^{-1}\right)\left(B:base,proton\;acceptor\right)$$

Carbamic acid mechanism (type III)

5 $${CO}_{2}+{RNH}_{2}⇄RNHCOOH\left(E_{a}=∼14-41\;kcal{\;mol}^{-1}\right)$$

6 $$RNHCOOH+{RNH}_{2}⇄RNH{COO}^{-}+{{RNH}_{3}}^{+}$$

To investigate the chemical species involved in CO_2_ adsorption under different temperature and humidity conditions, in situ IR experiments were performed via diffuse reflectance infrared spectroscopy (DRIFTS). Figure 6 shows the IR spectra for 40 wt %-PEI/ePTFE/silica during CO_2_ exposure for initial 60 min under dry (0% RH) and humid (50% RH) conditions at – 20 °C, 5 and 35 °C. The peak assignments are summarized in Table 2. The overall patterns of IR peaks observed under dry and humid conditions were similar, without significant new peaks appearing in the humid conditions. A weak band at 1692 cm^–1^ assigned to hydrogen-bonded carbamic acid only appeared under dry conditions (Figure 6a–c) and disappeared in humid conditions (Figure 6d,e), likely due to rapid conversion to ammonium carbamate. Recent studies suggest that water vapor promotes the conversion of carbamic acid intermediates to ammonium carbamate by enhancing proton transfer between amine chains, thereby increasing carbon dioxide uptake under humid conditions.^32,33^

**Figure 6:**
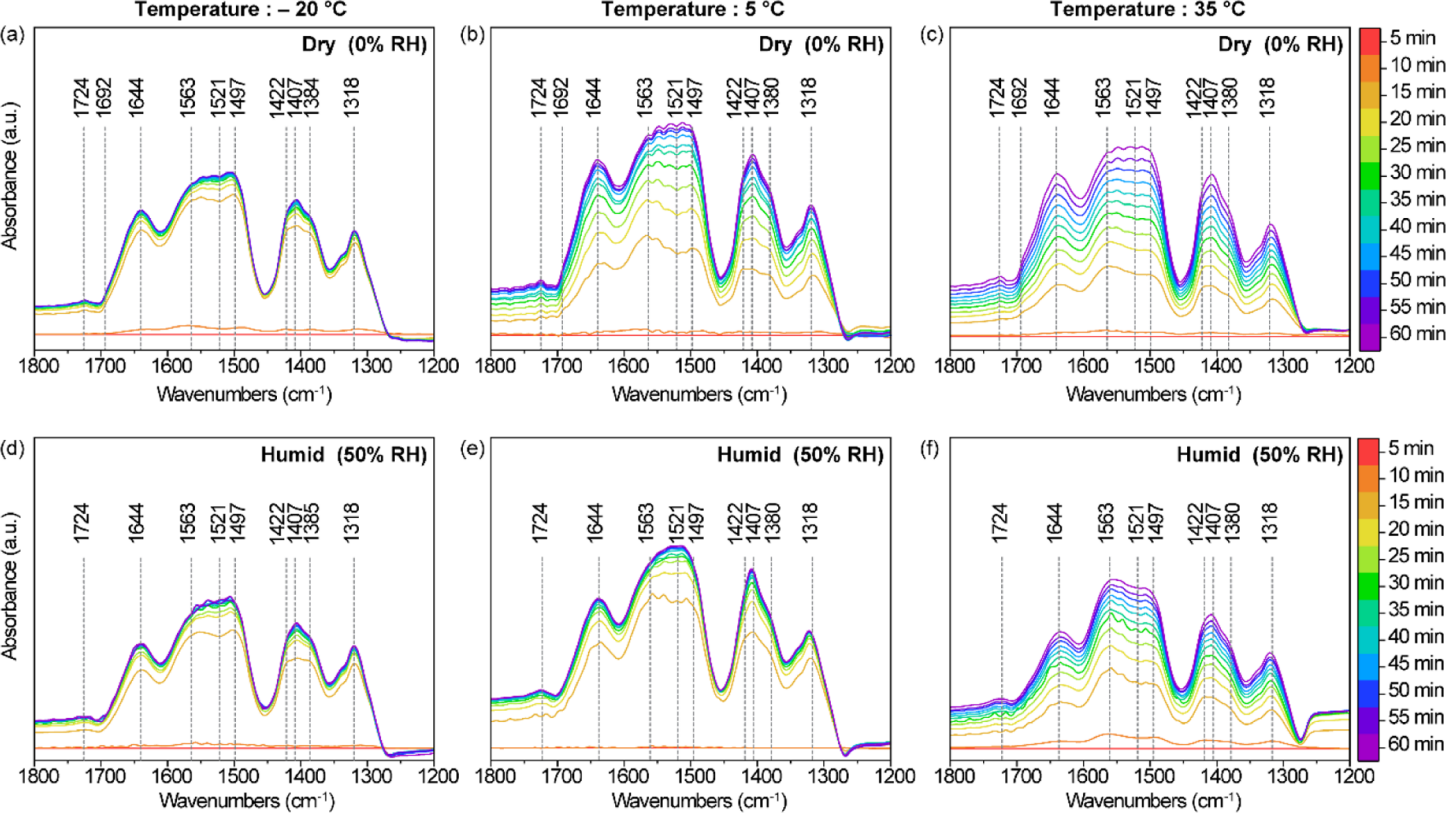
In situ IR spectra of 40 wt %-PEI/ePTFE/silica during CO_2_ exposure for 1 h under dry and humid conditions at various temperatures. (a–c) show spectra under dry conditions at (a) –20 °C, (b) 5 °C, and (c) 35 °C. (d–f) show spectra under 50% RH conditions at (d) –20 °C, (e) 5 °C, and (f) 35 °C. The related peak assignment is shown in Table 2.

**Table 2:** In situ IR Spectra Assignments for Absorbed CO_2_

wavenumber (cm^–1^)	assignment	species	refs
1724	C= O str	bound carbamate	^33^
1692	C= O str	carbamic acid (hydrogen-bonded), dimer carbamic acid	^32,33,51^
1628–1644	NH_3_^+^ def	primary ammonium ions	^32,33,51^
1563–1549	COO^–^ str	carbamate	^32,33,51^
1521	NH_3_^+^/NH_2_^+^ def	primary/secondary ammonium ions	^32^
1486–1497	COO^–^ str	carbamate	^32,33,51^
1407	NH_2_^+^ def	secondary ammonium ions	^32^
1380	COO^–^ str/NCOO^–^ skeletal vib	carbamate	^32,33^
1318	NCOO^–^ skeletal vib	carbamate	^32,51^

Additionally, since the 1521 cm^–1^ band represents the NH_3_^+^/NH_2_^+^ deformation of primary and secondary ammonium ions, we calculated the time-dependent 1521 cm^–1^/1563 cm^–1^ ratio to track the growth of ammonium relative to carbamate (1563 cm^–1^, COO^–^ asymmetric stretch). As shown in Figure S9a–f, the intensity at 1521 cm^–1^ and 1563 cm^–1^ increases with similar trends under both dry and humid conditions. However, when comparing the intensity ratio of the two peaks (1521 cm^–1^/1563 cm^–1^), the values under humid conditions are higher than those under dry conditions at – 20 and 5 °C, whereas at 35 °C, this ratio under humid conditions is lower. This is also observed at 30 wt %-PEI/ePTFE/silica (Figure S10) and 50 wt %-PEI/ePTFE/silica (Figure S11). This suggests that adsorbed water effectively enhances the mobility of PEI at lower temperatures (−20 and 5 °C) and higher PEI loadings and allows the carbamic acid intermediate to stabilize more quickly into ammonium carbamate. This is particularly evident at 5 °C. Additional DRIFTS data collected at 20% RH for the 5 °C condition show nearly identical 1521 cm^–1^/1563 cm^–1^ results to that at 50% RH (Figure S9), indicating that the preadsorbed water under low humidity is already sufficient to facilitate water-assisted proton transfer between amine chains. At – 20 °C, the temperature is so low that the increase in the mobility of the aminoploymer is limited during the first hour, whereas at the moderate temperature of 5 °C, the NH_3_^+^/NH_2_^+^ deformation under humid CO_2_ conditions increases rapidly, within ∼30 min, compared to dry conditions. On the other hand, at high temperatures (35 °C), where PEI chains are already mobile, excessive water may delay the formation of ammonium carbamate by blocking access to the amine site or inhibiting CO_2_ uptake through competitive adsorption.

To further support the enhancement of PEI mobility by water, we analyzed the IR band ratios at 2516 cm^–1^ and 3030 cm^–1^, which indirectly indicate hydrogen bonding within the ammonium-carbamate network. The band at 3030 cm^–1^ corresponds to the N–H stretching of NH_3_^+^ or carbamate species and is relatively insensitive to hydrogen bonding changes. In contrast, the band at 2516 cm^–1^ is attributed to the hydrogen-bonded NH_3_^+^ groups. Therefore, a decrease in 2516 cm^–1^/3030 cm^–1^ ratio reflects a diminished hydrogen bonding of ammonium-carbamate species.^51^ As shown in Figure S12, this ratio increases across all temperatures under humid conditions (50% RH) for 40 wt %-PEI/ePTFE/silica. This suggests that water acts as a bridging agent forming hydrogen bonds between adsorbed CO_2_ species and neighboring amines while simultaneously disrupting the hydrogen bonding within the PEI polymer, thereby increasing PEI chain flexibility. These measurements were performed within the first hour after exposure and therefore provide insight into early stage mobility changes rather than equilibrium behavior.

As the PEI loading increased from 30 to 50 wt %, both the overall CO_2_ capacity in most conditions and the maximum CO_2_ capacity of each sample improved, reaching 1.77 mmol/g (30 wt %-PEI), 2.61 mmol/g (40 wt %-PEI), and 3.49 mmol/g (50 wt %-PEI), respectively. These trends are clearly indicated in Figure S13a,c, where the graphs are standardized with the same contour range of 0.41 to 3.5 mmol/g to enable direct comparison. This suggests that a higher PEI loading facilitates increased active site availability for CO_2_ adsorption. However, the amine efficiency, which represents the effectiveness of the amine sites, showed a maximum value of 0.29 across all materials under certain conditions (Figure S13d–f). While environmental changes influenced all samples similarly, higher PEI loading samples demonstrated greater sensitivity to humidity at low temperatures (−20 °C). This indicates that higher PEI content sorbents are more moisture sensitive, likely due to water-enhanced amine availability, especially in cold environments. When focusing only on absolute CO_2_ capacity, PEI/ePTFE/silica materials with higher PEI loading proved to be more advantageous across most climatic conditions.

The contour map generated for a single sample provides valuable insights but has limitations in fully evaluating the influence of PEI loading on the temperature and humidity interactions. Therefore, additional analyses were performed using the contour maps discussed in the subsequent sections, which explore the independent effects of PEI loading, humidity, and temperature.

### Effect of Humidity and PEI Loading on CO_2_ Adsorption at Fixed Temperature

The effect of humidity under different temperatures is shown in Figure 7a–c and d–e, which present the CO_2_ capacity and amine efficiency, respectively. In Figure 7a (−20 °C), the sorbents under dry conditions show a dark blue color in the contour map, which corresponds to a relatively low CO_2_ capacity (0.45–0.74 mmol/g). A gradual increase in the CO_2_ capacity is observed as the RH increases up to 80%. In contrast, Figure 7b (5 °C) shows a slightly higher capacity (0.83–0.92 mmol/g, indicated by a lighter blue) under dry conditions compared to −20 °C, and the capacity shows a continuous increasing trend with increasing RH. However, for the sorbents loaded with 40 and 50 wt %-PEI, the capacity peaks before 80% RH and then decreases. Specifically, the 50 wt %-PEI/ePTFE/silica shows a significant decrease in the CO_2_ capacity from 3.38 mmol/g (50%RH) to 2.89 mmol/g (80% RH) at higher humidity. Interestingly, the effect of humidity appears to be minimal at 35 °C (Figure 7c). The contour plots show little color change across different humidity levels, indicating limited changes in the CO_2_ capacity. The differences in capacity between 0% RH and 80% RH are relatively small: −0.18 mmol/g (30 wt %-PEI), −0.12 mmol/g (40 wt %-PEI), and 0.22 mmol/g (50 wt %-PEI), with some cases even showing a slight decrease. While humidity has a reduced impact on the CO_2_ capacity at elevated temperatures, this does not imply that the role of water is negligible.

**Figure 7:**
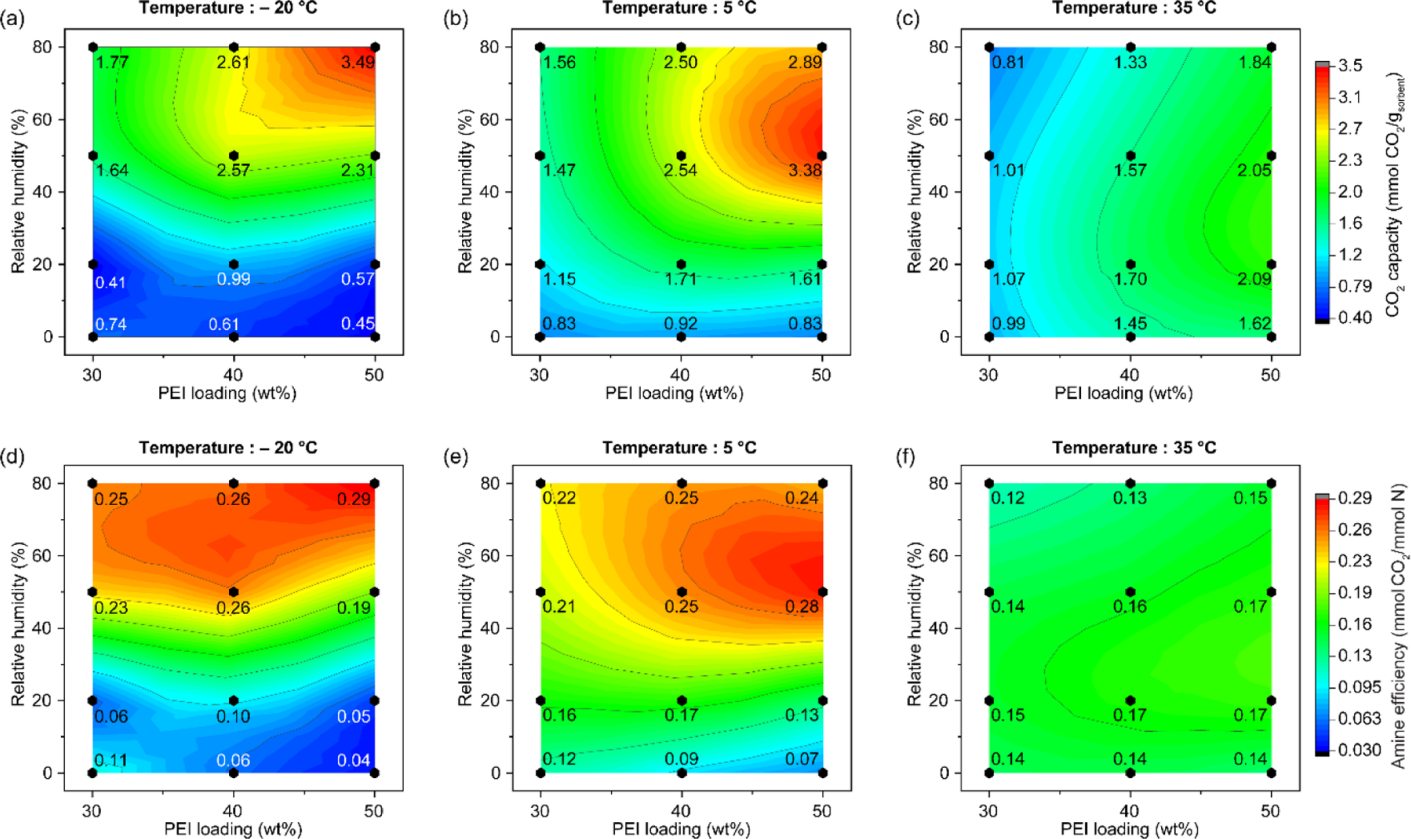
Contour maps of CO_2_ capacity at different PEI loadings and different relative humidities at (a) –20 °C, (b) 5 °C, and (c) 35 °C using the fixed-bed system. All contour maps were plotted using the same capacity range (0.40–3.5 mmol/g). (d–f) Contour maps of amine efficiency at different PEI loadings and different RH at (d) –20 °C, (e) 5 °C, and (f) 35 °C. All contour maps were plotted using the same capacity range (0.03–0.29 mmol/g).

The effect of humidity is more evident upon examination of the amine efficiency, as shown in Figure 7d–f. At –20 °C (Figure 7d), the contours increase almost linearly with an increasing humidity, regardless of PEI loading. This trend is supported by the in situ IR spectra (Figure 6), which show that the carbamic acid intermediate is rapidly converted to ammonium carbamate, likely via a water-assisted proton transfer. At 5 °C (Figure 7e), the effect of humidity appears to depend more on the PEI loading. Samples with higher PEI loading show a more pronounced increase in amine efficiency with humidity compared to those with a lower PEI loading. This suggests that water not only shifts the reaction pathway toward water-assisted proton transfer but also enhances PEI polymer chain mobility and diffusivity.^42,52^ So, these humidity effects are clearly observed in our study at – 20 and 5 °C. On the other hand, at 35 °C (Figure 7f), the effect of humidity on amine efficiency is negligible. This suggests that the mobility of PEI is already sufficiently high at higher temperatures so that the additional effect of water is reduced. In addition, due to the elevated water vapor concentration in the external atmosphere (Table S4) at higher temperature, water may more effectively kinetically compete with CO_2_ for adsorption sites, which may hinder CO_2_ sorption under warm, high humidity conditions.

Competitive adsorption between water and CO_2_ exhibits both thermodynamic and kinetic dependencies that should be considered separately. As shown in the single-component water sorption isotherm (Figure 8a, measured by VSTAR, Anton Paar), the adsorbed water amine efficiency (mmol of H_2_O/mmol of N) remained constant across the sorbents at each RH level, less affected by the PEI loading and temperature. Thus, the thermodynamics of water sorption in the presaturation step primarily depends on the RH, and not on the temperature. However, the kinetics of water uptake are strongly temperature-dependent, which significantly influences the flux of water toward the sorbent binding sites. As shown in Figure S14, the slope of the water uptake profile shows that faster rates of water uptake were observed at higher temperature and at higher RH. The higher flux of water can accelerate site blocking in competitive adsorption with CO_2_, affecting the subsequent water–CO_2_–amine interaction dynamics.

**Figure 8:**
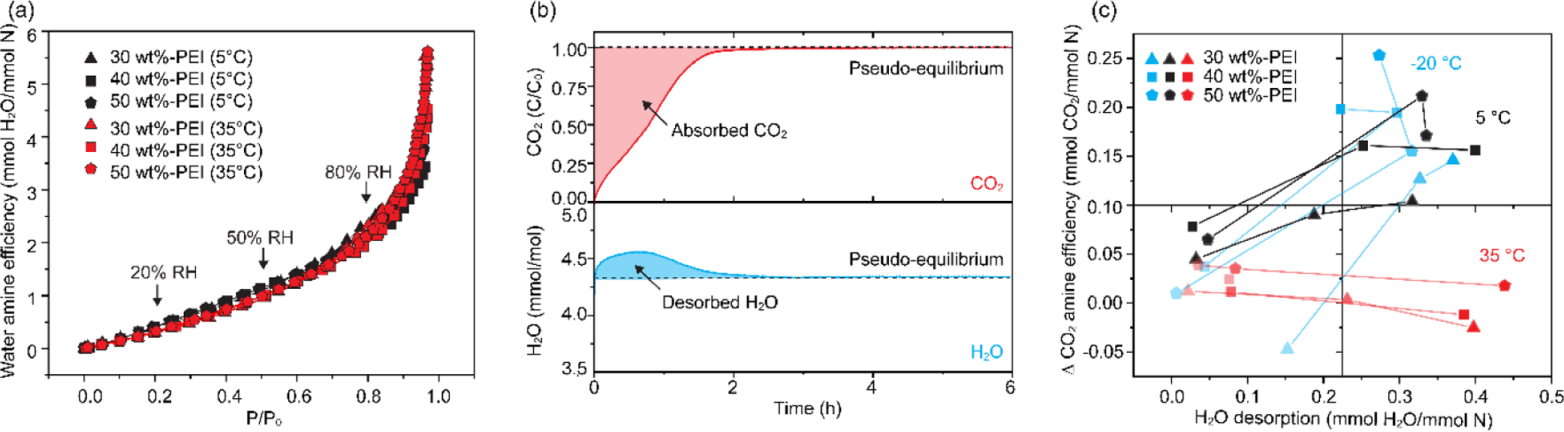
(a) Water adsorption isotherms of 30, 40, and 50 wt %-PEI/ePTFE/silica sorbents at 5 and 35 °C. (b) Representative normalized CO_2_ uptake profiles (*C*/*C*_0_) and corresponding water desorption profiles (mmol/mol) during the humid CO_2_ sorption step at 5 °C and 50% RH using the 30 wt-% PEI/ePTFE/silica sorbent. (c) Relationship between water desorption (mmol H_2_O/mmol N) and increased CO_2_ amine efficiency relative to dry conditions (mmol CO_2_/mmol N) under varying PEI loading levels (30–50 wt %), relative humidities (20–80% RH), and temperatures (−20 to 35 °C).

During humid CO_2_ adsorption, partial desorption of preadsorbed water was consistently observed for all conditions and sorbents (Figure 8b). This is roll-up behavior,^53,54^ wherein the outlet H_2_O concentration exceeds the inlet value upon introduction of CO_2_ (Figure S15d). The stabilization points of water release and CO_2_ adsorption overlap closely, suggesting that water and CO_2_ compete for access to the amine sites during the adsorption process. Moreover, both water desorption and the CO_2_ uptake profiles stabilize more rapidly at higher temperatures (Figure S16). These results suggest that polymer chain mobility and water redistribution kinetics critically affect the timing and efficiency of CO_2_ chemisorption. At –20 °C, despite limited mobility of both water and polymer chains, preadsorbed water molecules effectively facilitate CO_2_ chemisorption by assisting proton transfer reactions, resulting in measurable capacity increases under humid conditions. As the temperature increases to 5 °C, moderate mobility creates an optimal kinetic window where water molecules can simultaneously assist proton transfer and provide modest PEI plasticization before competitively occupying the reactive amine sites. In contrast, at 35 °C, rapid water redistribution promotes stronger competition with CO_2_ for amine sites, limiting CO_2_ capacity gains from humidity. In the coadsorption experiments at – 20 °C (Figure S15a), a unique readsorption behavior is observed under 50% (253 ppm water) and 80% (1013 ppm water) RH conditions. The outlet CO_2_ concentration initially rises sharply and approaches the inlet concentration (400 ppm), similar to dry conditions, but it subsequently decreases again over several hours with bimodal adsorption. This bimodal behavior can be understood as a kinetic two-step process dominated by fluxes at relatively low water concentrations. The initial uptake reflects dry-like adsorption, followed by a delayed, water-assisted CO_2_ chemisorption phase that emerges once sufficient water has been adsorbed and redistributed within the sorbent, thereby enhancing polymer mobility. This adsorption transition resembles the dual-shock breakthrough profile observed in amine-functionalized MOFs, which arises from transitions between distinct adsorption mechanisms.^55^ At higher temperatures of 5 and 35 °C, where water concentrations are higher and polymer/molecular mobility is sufficient, water redistribution occurs more rapidly, and CO_2_–H_2_O interaction appears immediately. As a result, a distinct bimodal profile is not observed under these conditions. Together, these observations emphasize that competitive adsorption between CO_2_ and water is not an equilibrium phenomenon but is significantly shaped by kinetic factors, including temperature, hydration state, and polymer mobility.

This interpretation is further supported by the correlation shown in Figure 8c, which shows the relationship between the desorbed water amine efficiency (mmol H_2_O/mmol N) and the change in CO_2_ amine efficiency between humid and dry conditions (ΔCO_2_ amine efficiency, AE = AE_humid_–AE_dry_). Data points corresponding to 20%, 50%, and 80% RH are connected to emphasize the trend with each humidity level. At –20 °C (blue) and 5 °C (black), the ΔCO_2_ amine efficiency exhibited a clear positive correlation with the amount of water desorbed, which increased with RH. This suggests that the ΔCO_2_ amine efficiency is enhanced at lower temperatures due to improved accessibility of CO_2_ to amine sites, either by water desorption or by water-assisted proton transfer, thereby facilitating the rapid conversion of carbamic acid to ammonium carbamate. In contrast, at 35 °C (red), this correlation was not observed. The ΔCO_2_ amine efficiency remained nearly constant regardless of the amount of desorbed water. This indicates that at elevated temperatures, the intrinsic thermal mobility of PEI may already be sufficient to enable CO_2_ adsorption, thereby reducing the beneficial effect of water.

### Effect of Temperature and PEI Loading on CO_2_ Adsorption at Fixed Relative Humidity

Contour maps of the CO_2_ capacity (Figure 9a–d) and amine efficiency (Figure 9e–h) were plotted to illustrate the effect of temperature at each humidity condition. Under dry conditions (0% RH), the highest capacity was observed for the 50 wt %-PEI/ePTFE/silica at 35 °C. As RH increased to 20%, the overall capacity improved, but the maximum still occurred at 35 °C with 50 wt %-PEI/ePTFE/silica. The maximum CO_2_ capacity at higher RH was observed for the 50 wt %-PEI/ePTFE/silica at 5 °C under 50% RH and at –20 °C under 80% RH. The highest capacity was observed in the 50 wt %-PEI/ePTFE/silica, consistent with the TGA results (Figure 2), showing that higher PEI content provides more amine sites, thereby enhancing the CO_2_ capacity, regardless of temperature. However, the maximum capacity shifts to lower temperatures with higher RH, which may be due to the synergistic effect between water and amine groups under cold and humid conditions, where enhanced PEI mobility and promotion of water-assisted proton transfer can contribute to improved CO_2_ uptake.

**Figure 9:**
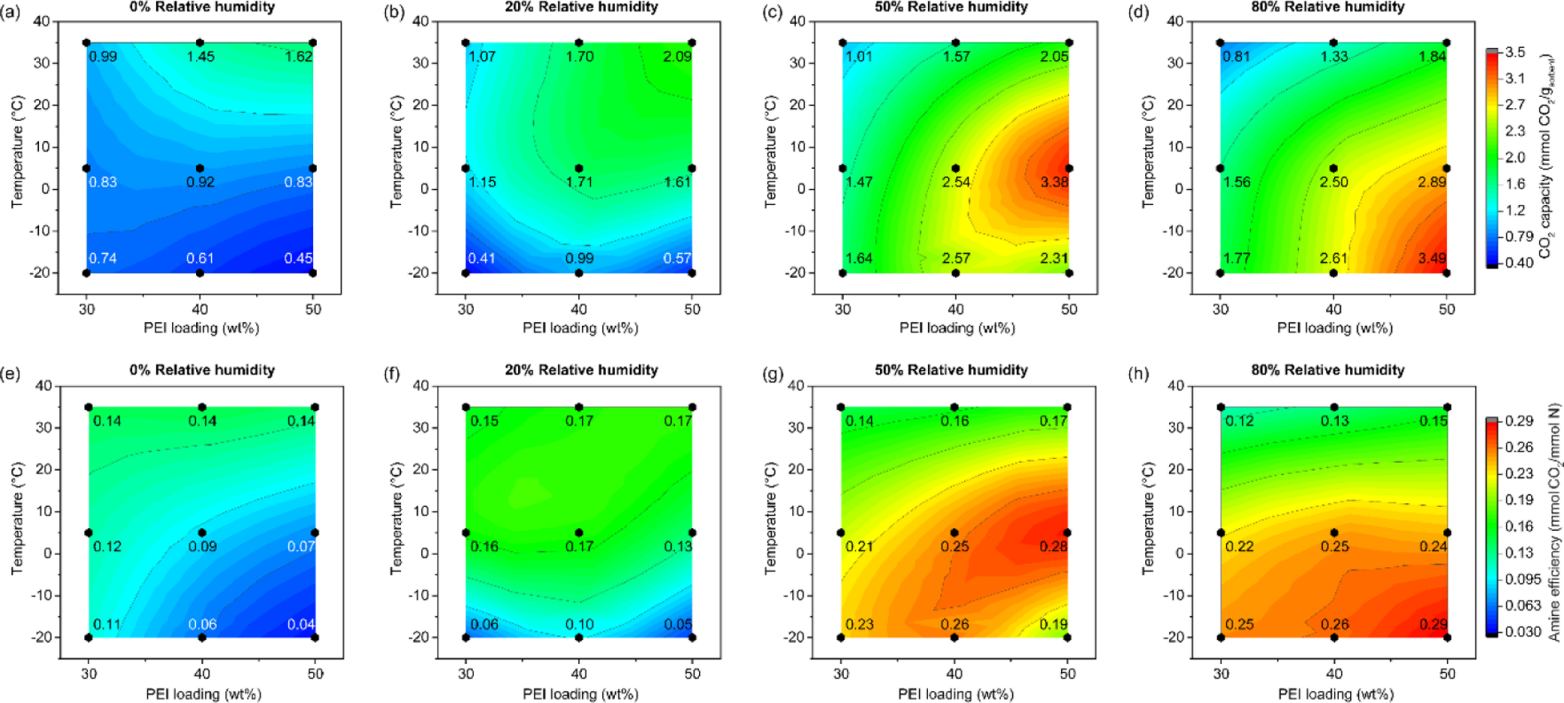
(a–d) Contour map of CO_2_ capacity at different PEI loadings and different temperatures at (a) 0% RH, (b) 20% RH, (c) 50% RH, and (d) 80% RH using the fixed-bed system. All contour maps were plotted using the same capacity range (0.40–3.5 mmol/g). (e–h) Contour map of amine efficiency at different PEI and different temperatures: (e) 0% RH, (f) 20% RH, (g) 50% RH, and (h) 80%. All contour maps were plotted using the same capacity range (0.03–0.29 mmol/g).

Amine efficiency plots (Figure 9e–h) provide additional insight. Under dry conditions (0% RH, Figure 9e), the amine efficiency was similar across 30–50 wt %-PEI/ePTFE/silica at 35 °C but dropped significantly at lower temperatures, particularly for 40 and 50 wt % PEI. This suggests that lower temperatures reduce PEI chain mobility, limiting access of CO_2_ to amine sites in the absence of water. At 20% RH (Figure 9f), the amine efficiencies at 5 and 35 °C were similar to the dry 35 °C values for all PEI loadings but still remained lower at – 20 °C. The presence of water at this low humidity is sufficient to enhance polymer mobility and facilitate proton transfer between amine chains, restoring efficiency comparable to that achieved under dry, high-temperature conditions. As RH increased to 50% (Figure 9g) and 80% (Figure 9h), the amine efficiency began to increase at 5 °C and −20 °C (0.19–0.29 mmol CO_2_/mmol N) compared to 35 °C (0.12–0.17 mmol CO_2_/mmol N). These additional gains at higher RH are likely due to cumulative effects of enhanced water-assisted CO_2_ transport and increased proton transfer between amine chains, which are important for enhancing rates under cold and humid conditions. Overall, these results indicate that RH influences CO_2_ adsorption through the combined effects of polymer and adsorbate mobility as well as water-assisted reaction pathways, with increasing humidity amplifying the overall contribution of these mechanisms. Although the 50 wt %-PEI/ePTFE/silica sample consistently showed the highest CO_2_ capacity, sorbent performance in practical DAC applications depends not only on capacity but also on sorption kinetics. The following section evaluates the kinetic performance of each sorbent material.

### Kinetics of CO_2_ Adsorption

High adsorption rates of target gas molecules are critical for the economic viability of adsorption-based separations, particularly in processes such as DAC. Rapid adsorption kinetics shortens cycle times and enhances overall process productivity. Over the years, a variety of kinetic models have been proposed to describe the temporal evolution of the adsorbate loading on solid sorbents. Among the most widely applied are the Lagergren’s pseudo first-order, pseudo second-order, Avrami,^56–58^ and intraparticle diffusion (IPD)^59,60^ formulations, each reflecting different mechanistic assumptions about how adsorbate molecules interact with and penetrate the adsorbent. Among these models, the IPD model is particularly useful because it yields direct insight into the distinct mass-transfer regimes such as boundary-layer resistance, pore diffusion, and equilibrium without invoking complex geometry or multiparameter fits. This model is based on Fick’s second law of diffusion and assumes the rate of adsorption is controlled by intraparticle diffusion, which is a suitable assumption for aminopolymer-based sorbent systems,^61,62^ and expressed as

$$\frac{q\left(t\right)}{q_{final}}=k_{p}t^{1/2}+C$$

where *k*_*p*_ is the normalized IPD rate constant (min^–1/2^) and C is a constant representing the intercept. IPD model’s single *t* ^1/2^ dependency and straightforward rate constants facilitate rapid comparison of diffusional behavior across diverse sorbents, making trend analysis transparent and reproducible. According to this model, the *q*(*t*)­vs.*t* ^1/2^ plot should yield a straight line with a zero y intercept if the intraparticle diffusion is the only rate-determining step. Nevertheless, multilinearity is commonly observed, most often appearing as three steps: the first step corresponding to external surface diffusion, the second step to intraparticle diffusion, and the third step to the final equilibration step.^59^ Here, we seek to use the IPD model to understand how H_2_O affects the adsorption kinetics of CO_2_ on PEI under various conditions in the laminate system. In the laminate system, there are five processes involved in CO_2_ mass transport and adsorption: external gas phase, laminate macropore phase (interparticle phase), silica particle pore phase diffusions, diffusion in the PEI matrix (intraparticle phases), and final equilibration (reaction/sorption). When these processes are applied to the IPD model, the first step corresponds to both external and macropore phase diffusion (fast mass transport), the second step involves diffusion inside the silica pores, and PEI matrix/reaction with amines (rate-limiting step).^62,63^

Figures 10 to 11 present the CO_2_ uptake curves vs square root of time of CO_2_ uptake under 12 different conditions within the first 625 min of adsorption. Under cold and humid conditions, CO_2_ adsorption slowed considerably due to limited mobility of PEI chains, and sorbents under these conditions did not reach equilibrium during this period. The initial slope of the uptake curve representing the early adsorption rate is generally similar across all PEI loadings. However, at low temperatures and high humidity (−20 °C/50–80% RH, 5 °C/80% RH), the 50 wt %-PEI/ePTFE/silicas shows significantly slower uptake behavior. The 50 wt %-PEI/ePTFE/silica showed the highest capacity in equilibrium-based comparisons, but it reached equilibrium much more slowly and had lower CO_2_ capacity within short time frames, demonstrating the mass transfer limitation by an excess of PEI chains. This mass transfer limitation by PEI chains is further supported by DSC analysis, as shown in Figure S17. The glass transition temperature (*T*_g_) of the PEI/ePTFE/silica sorbents decreases with increasing water uptake, indicating an enhanced segmental mobility of the polymer chains. Before presaturation, the 50 wt %-PEI/ePTFE/silica exhibits a lower *T*_g_ compared to 30 wt %-PEI/ePTFE/silica because of the presence of more bulk-phase PEI domains with reduced interaction with the silica surface.^64,65^ However, after presaturation under humid N_2_ flow, the 30 wt %-PEI sorbent shows the largest reduction in *T*_g_, whereas the 50 wt %-PEI sorbent shows the smallest change. This trend suggests that a greater fraction of PEI interacts directly with moisture in lower PEI loading materials and results in a more pronounced plasticization and increased chain flexibility. In contrast, the dense PEI network in the 50 wt %-PEI sorbent limits plasticization by water and thus shows less reduction in *T*_g_. These findings are consistent with the proposed mechanism in which water enhances PEI mobility and diffusion, especially in sorbents with lower PEI loading.

**Figure 10:**
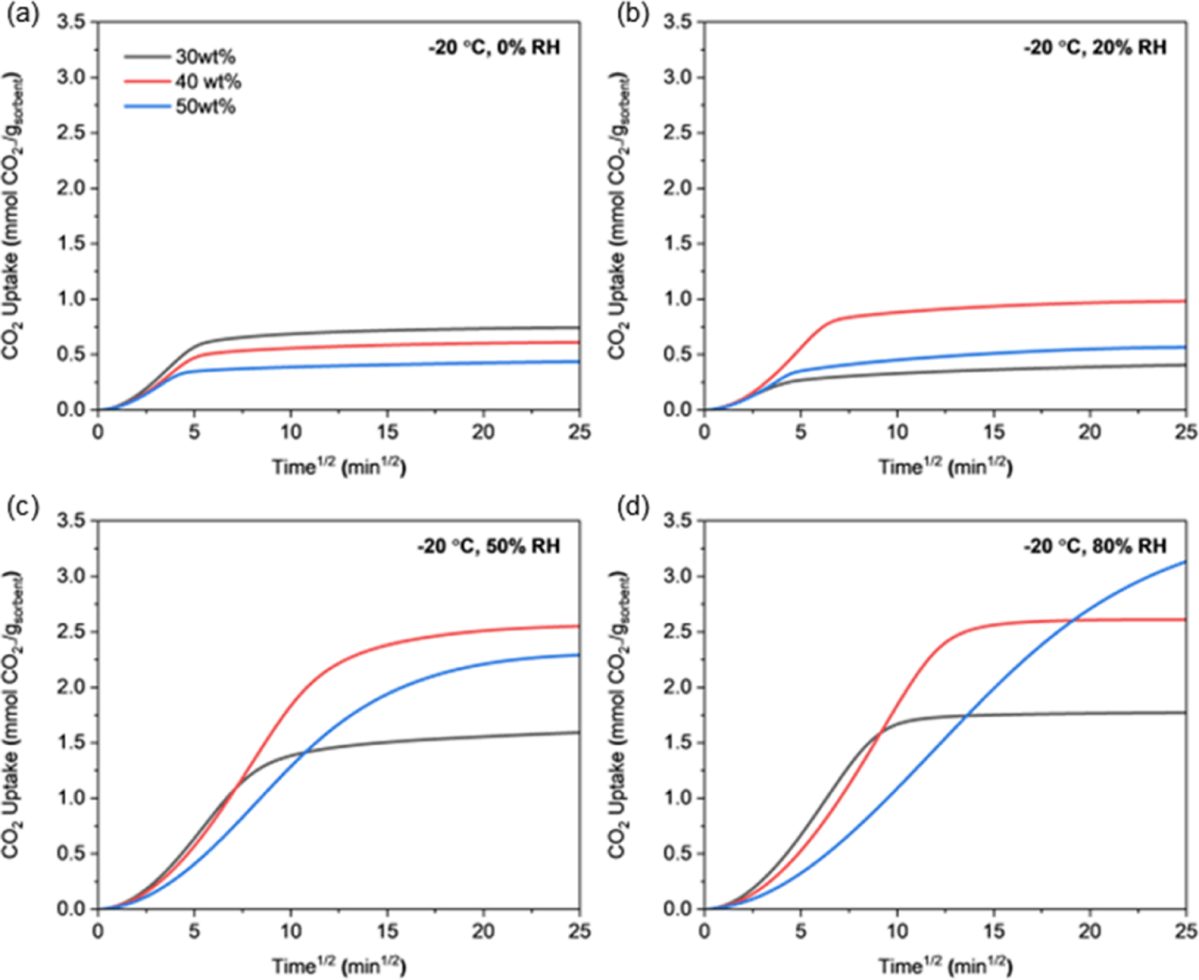
CO_2_ uptake profiles of 30, 40, and 50 wt %-PEI/ePTFE/silica sorbents at –20 °C and under varying relative humidities measured in the fixed-bed system. (a), 0% RH, (b) 20% RH, (c) 50% RH, and (d) 80% RH.

**Figure 11:**
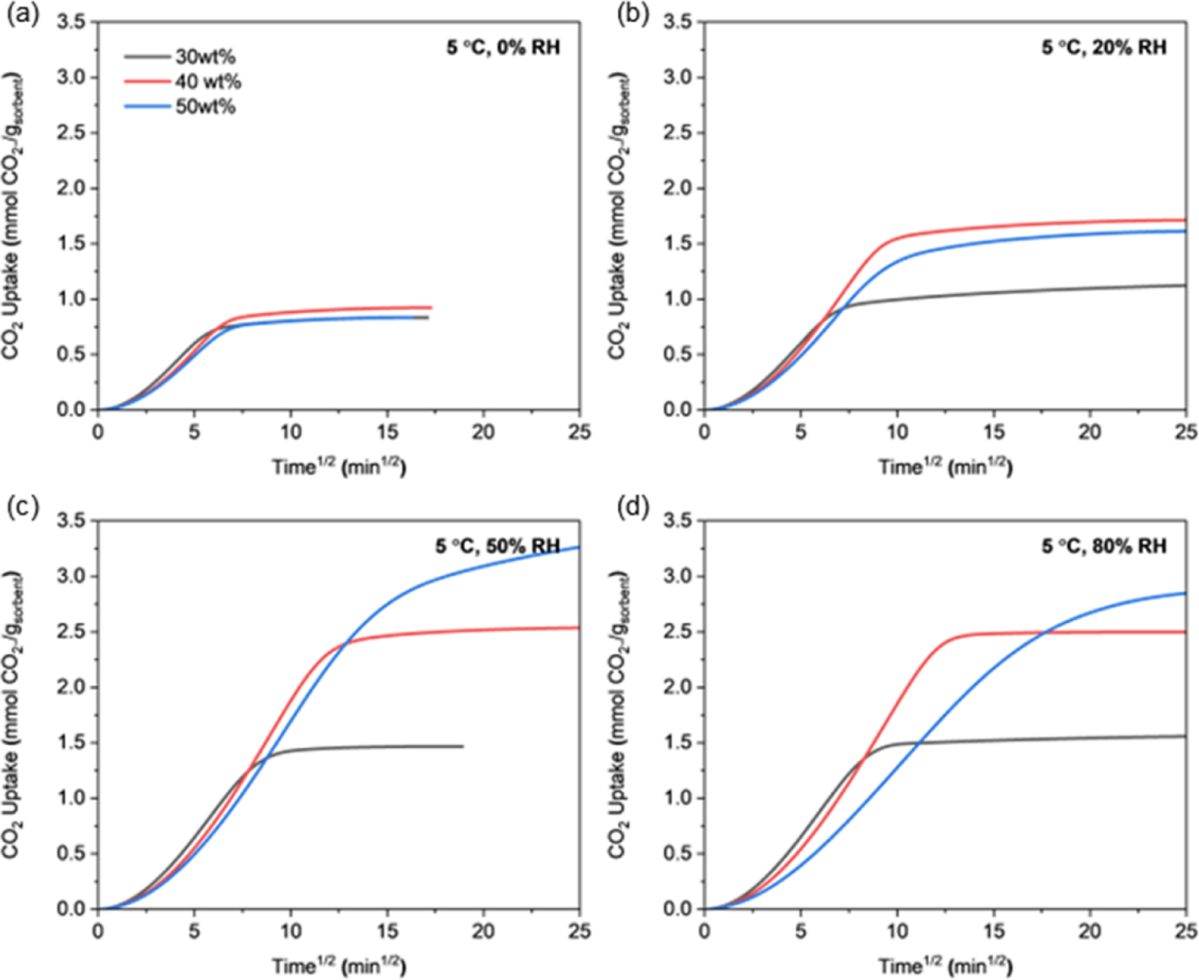
CO_2_ uptake profiles of 30, 40, and 50 wt %-PEI/ePTFE/silica sorbents at 5 °C and under varying relative humidities measured in the fixed-bed system. (a) 0% RH, (b) 20% RH, (c) 50% RH, and (d) 80% RH.

**Figure 12:**
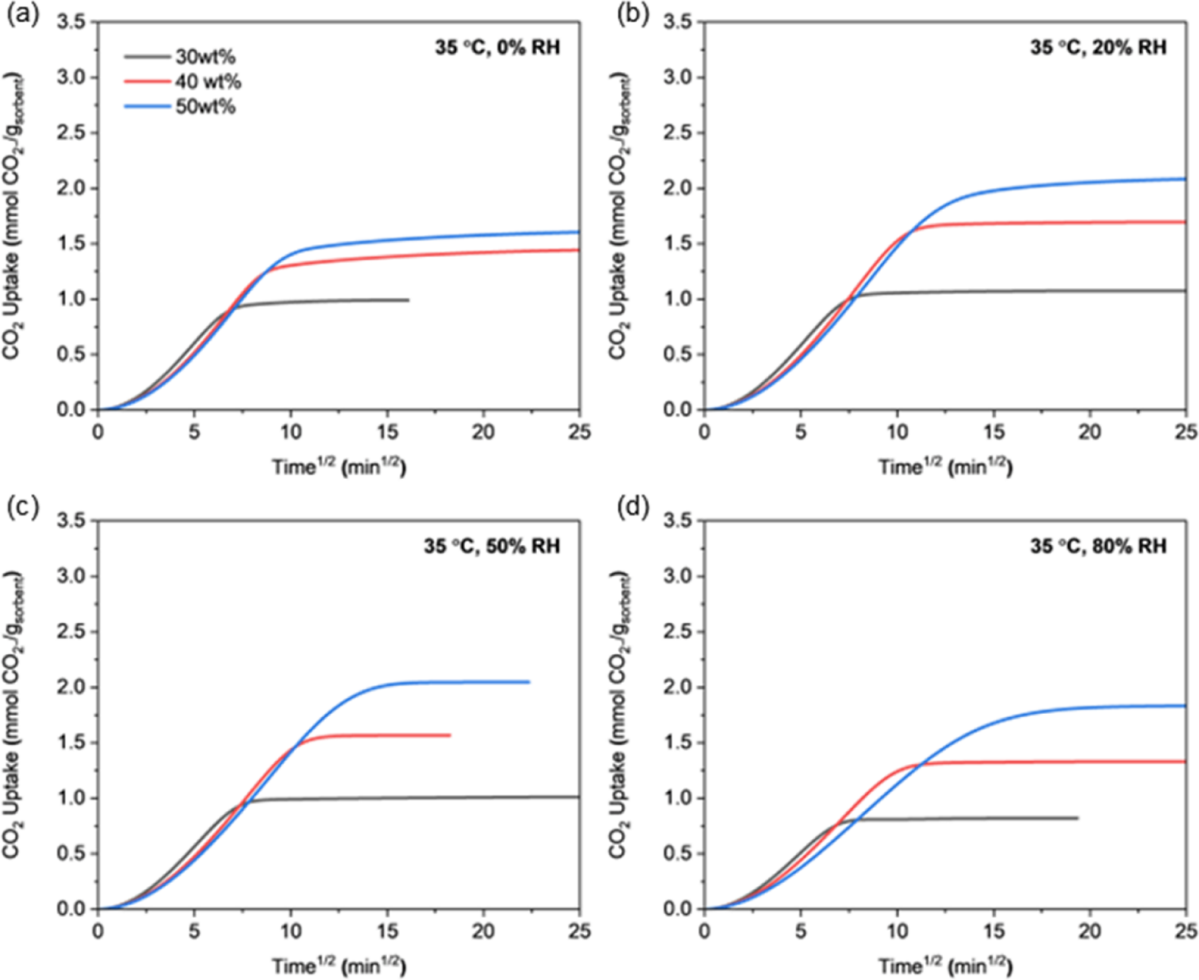
CO_2_ uptake profiles of 30, 40, and 50 wt %- PEI/ePTFE/silica sorbents at 35 °C and under varying relative humidities measured in the fixed-bed system. (a) 0% RH, (b) 20% RH, (c) 50% RH, and (d) 80% RH.

Since the sorbents were presaturated with water prior to CO_2_ introduction, water undergoes rearrangements or even replacement triggered by CO_2_ sorption. In contrast to water, which was preadsorbed, CO_2_ effectively diffuses into the silica pores where it competes with preadsorbed water for amine sites. As shown in Figure 8b, water desorption and CO_2_ adsorption occur simultaneously. Across all tested temperatures and humidities, increasing the PEI loading consistently decreased the diffusion rate. This is due to the presence of buried amine sites under a denser PEI network, which restricts CO_2_ transport. The slowdown is particularly pronounced at higher PEI loadings, low adsorption temperature, and high water uptake (−20 °C/50–80% RH, 5 °C/80% RH) due to dense PEI coatings inside the silica pores, reduced PEI chain mobility at lower temperature, and larger amount of preadsorbed water (impeding diffusion, ultimately slowing down the transport of CO_2_ molecules into the sorbent matrix).

Figure 13 shows the fitted IPD rate constants from the second step of adsorption obtained from sorbents with different PEI loadings under different adsorption conditions. The variations in rate constant values indicate that (i) adsorbed water generally decreases CO_2_ sorption kinetics and (ii) the effect of water on CO_2_ sorption kinetics varies with temperature. First, the observed decrease in the rate constant at higher relative humidities suggests that although water plasticizes the PEI chains, thereby releasing more amine sites for CO_2_ adsorption that were inaccessible under dry conditions, it may also lengthen the CO_2_ diffusion path inside the PEI matrix and/or impede its diffusion. It was also observed that the preadsorbed water desorbed after introducing the humid CO_2_ stream (Figure 8b), suggesting a competitive adsorption dynamic between water and CO_2_. The higher the PEI loading, the lower the rate constants, indicating that a greater mass transfer resistance is present in densely packed PEI inside the pores at high PEI loadings.

**Figure 13:**
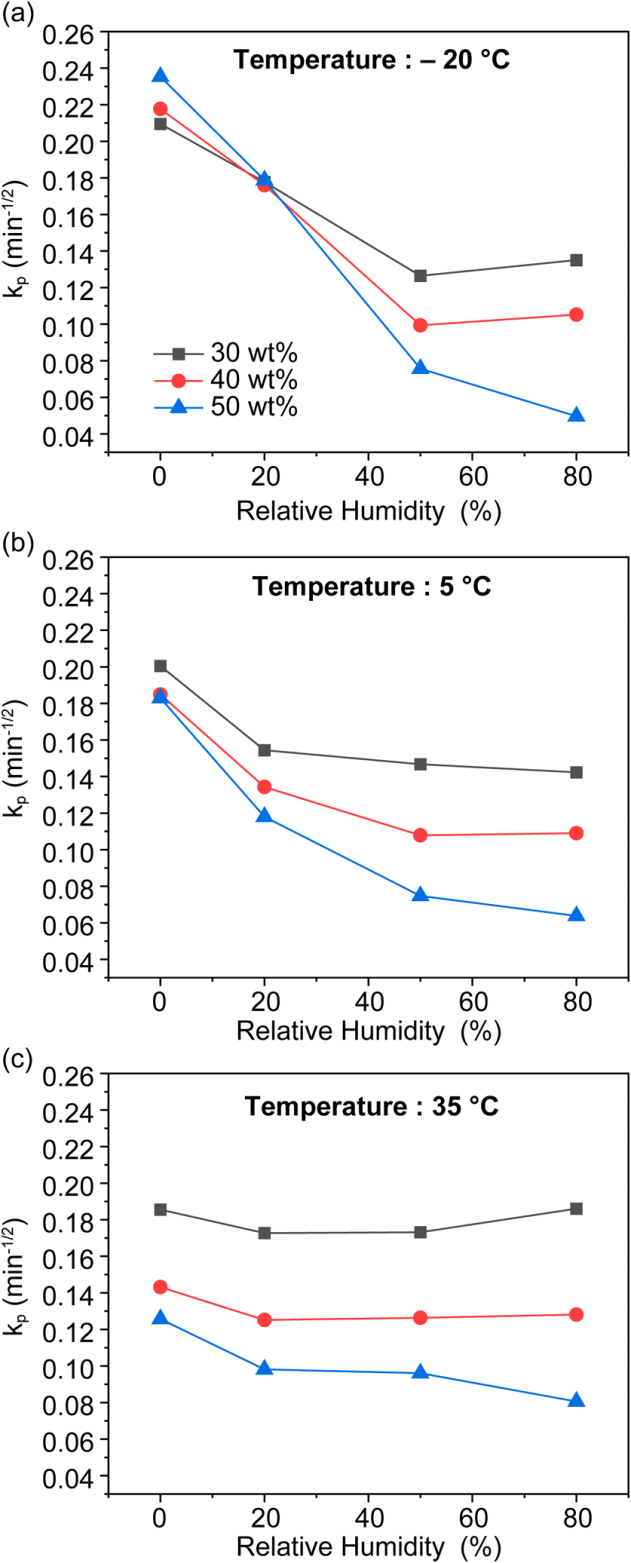
Fitted CO_2_ sorption IPD rate constants (the second step where intraparticle diffusion is dominant) for sorbents with different PEI loadings. (a) –20 °C, (b) 5 °C, and (c) 35 °C.

Furthermore, as shown in Figure S18, the water adsorption capacity is dominated by the RH and less affected by temperature, with prewater-saturated sorbents having similar water loadings and similar relative humidities. However, the decay of the rate constant as a function of RH seems to be less pronounced at higher temperatures. We postulate that at higher temperature, PEI chains are already mobile enough and adsorbed water did not effectively release previously inaccessible amine sites. Under these conditions, water is most likely to impede the mass transfer of CO_2_ and its reaction with amines.

While the previous analysis focused on the influence of PEI loading, temperature, and humidity on CO_2_ sorption kinetics, it is also important to understand how these effects change over time. Therefore, we investigated the time-dependent CO_2_ adsorption performance across different PEI loading. Figure 14 provides a visualization of how the optimal PEI loading that achieves the highest CO_2_ capacity shifts with sorption time (at 1, 2, and 3 h, all the way to pseudoequilibrium) under different temperature and RH conditions. Here, pseudoequilibrium was considered as the time when the CO_2_ capacity change remained below 0.1% within a 1 h period. Figure S19 shows the related absolute CO_2_ uptake profiles over time. After 1 h of humid CO_2_ exposure, the 30 wt %-PEI/ePTFE/silica generally exhibited the highest capacity at –20 °C, but at higher temperatures, the 40 wt %-PEI/ePTFE/silica began to perform better. After 2 h, the 40 wt %-PEI/ePTFE/silica has the highest capacity across most low temperature conditions (−20 and 5 °C), while the 50 wt %-PEI/ePTFE/silica gradually becomes dominant at 35 °C. After 3h, most conditions where both 40 wt %- and 50 wt %-PEI/ePTFE/silica previously showed similar performance disappeared, with the 50 wt %-PEI/ePTFE/silica clearly showing the dominant performance at 35 °C. Upon reaching a pseudoequilibrium, all 35 °C conditions and 80% RH conditions consistently showed the highest capacity. Although the 50 wt %-PEI/ePTFE/silica sorbent exhibited the highest theoretical capacity under many conditions, it took the longest time to reach equilibrium under various conditions. In contrast, the 40 wt %-PEI/ePTFE/silica sample consistently achieved equilibrium at a similar rate as the 30 wt %-sample while maintaining a higher capacity within the first 2 h (Figure 14). This suggests that the 40 wt %-PEI/ePTFE/silica provides the most balanced performance in terms of both the CO_2_ sorption rate and overall uptake capacity (Figure 14b).

**Figure 14:**
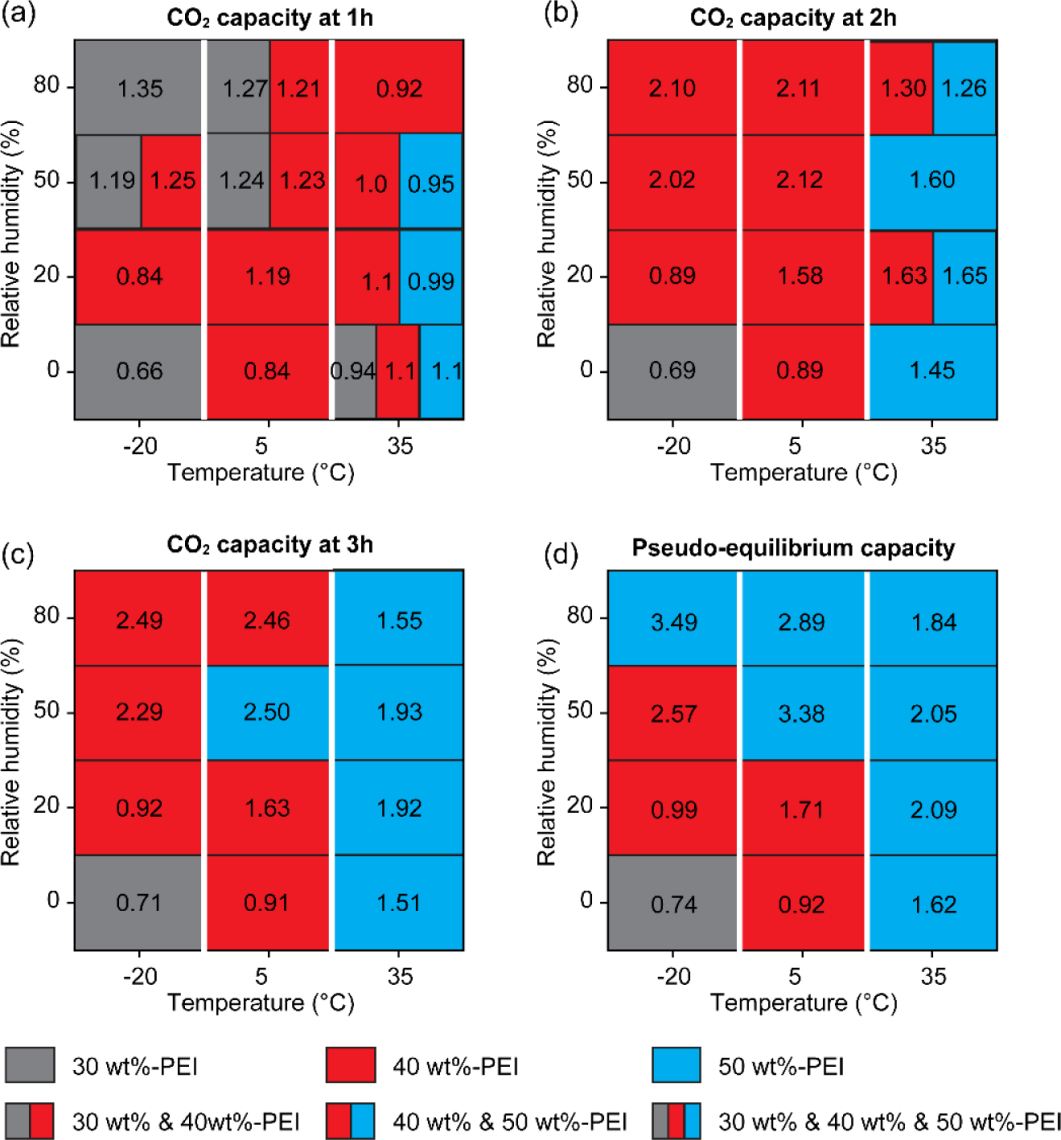
Heatmap showing the PEI loading that has the highest CO_2_ capacity (mmol CO_2_/g_sorbent_) at (a) 1 h, (b) 2 h, (c) 3 h, and (d) pseudoequilibrium under various temperature and RH conditions. The numerical values indicate the corresponding CO_2_ capacity for the optimal sorbent at each condition.

While the current study focused on material aspects and the adsorption performance as a function of conditions, the measured adsorption/desorption durations were not yet optimized, as one would in a scalable DAC process. For practical DAC implementation, strategies to enhance the sorption kinetics should be implemented, as discussed in our previous study.^62^ For example, higher gas flow rates would be used for adsorption to reduce the external mass transfer resistance. For the materials, optimization of the amine loading can be implemented with a focus on sorption kinetics, as the diffusion of CO_2_ inside the PEI matrix is most often the rate-limiting step in CO_2_ sorption, with lower amine loading sorbents generally exhibiting faster CO_2_ sorption kinetics in the temperature and humidity range studied (Figures 10–12, Figure S19). However, lowering the amine loading reduces the CO_2_ swing capacity at the same time, so the trade-off between the swing capacity and sorption kinetics should be balanced in the process optimization.

### Regeneration Temperature of Sorbents with Varied PEI Loading

Until this point, this study has focused on analyzing the effects of temperature, RH, and PEI loading on the CO_2_ adsorption performance. However, the energy required to regenerate the sorbents is another important factor influencing the operational cost-effectiveness of DAC systems. To evaluate the thermal behavior of CO_2_ release, temperature-programmed desorption (TPD) analysis was performed under N_2_ flow (100 mL/min) with a 1 h prepurge followed by a temperature ramp at 0.5 °C/min (Figure 15a). *T*_max_ and *T*_end_ increased continuously as the PEI loading increased (Figure 15b). In representative TPD profiles at 5 °C and 80% RH, the *T*_max_ and *T*_end_ increased from 62 and 87 °C (30 wt %-PEI) to 78 and 91 °C (40 wt %-PEI) and further to 85 and 98 °C (50 wt %-PEI). This trend is attributed to increased intraparticle diffusional resistance, specifically diffusional resistance inside the densely packed aminopolymers domains at higher loadings. As the PEI loading increases, the proportion of amine sites in the bulk region increases, and this creates longer and more tortuous CO_2_ diffusion pathways.^66^ As a result, CO_2_ molecules can repeatedly desorb and readsorb before escaping the sorbent matrix, delaying the completion of desorption. This phenomenon is clearly observed in Figure S20, which compares the TPD profiles of 30 and 50 wt %-PEI/ePTFE/silica across all conditions with diverse temperatures and humidities. While the *T*_end_ of CO_2_ desorption is barely affected by humidity in the 30 wt %-PEI sample, the 50 wt %-PEI sample shows noticeable desorption delays at higher humidity (Figure S20h–j). Moreover, the dense, bulk PEI matrix acts like a chromatographic column, where CO_2_ molecules undergo delayed and sequential desorption as they migrate through the internal domains. As a result, the two distinct desorption peaks observed between 40 and 60 °C at lower PEI loading (30 wt %-PEI) become broadened and merge into a single asymmetric peak at higher amine loadings. Also, this peak shifted to higher temperature in the 50 wt %-PEI sample, indicating suppressed peak separation and slower CO_2_ release. This contrasts with water desorption, which occurs at the same temperature range for both 30 wt %-PEI (Figure S20d–f) and 50 wt %-PEI (Figure S20k–m) samples, regardless of humidity. This suggests that water desorption is mainly determined by weak hydrogen bonding or physical adsorption and is not significantly affected by diffusion constraints or competitive adsorption kinetics, such as chemically adsorbed CO_2_.

**Figure 15:**
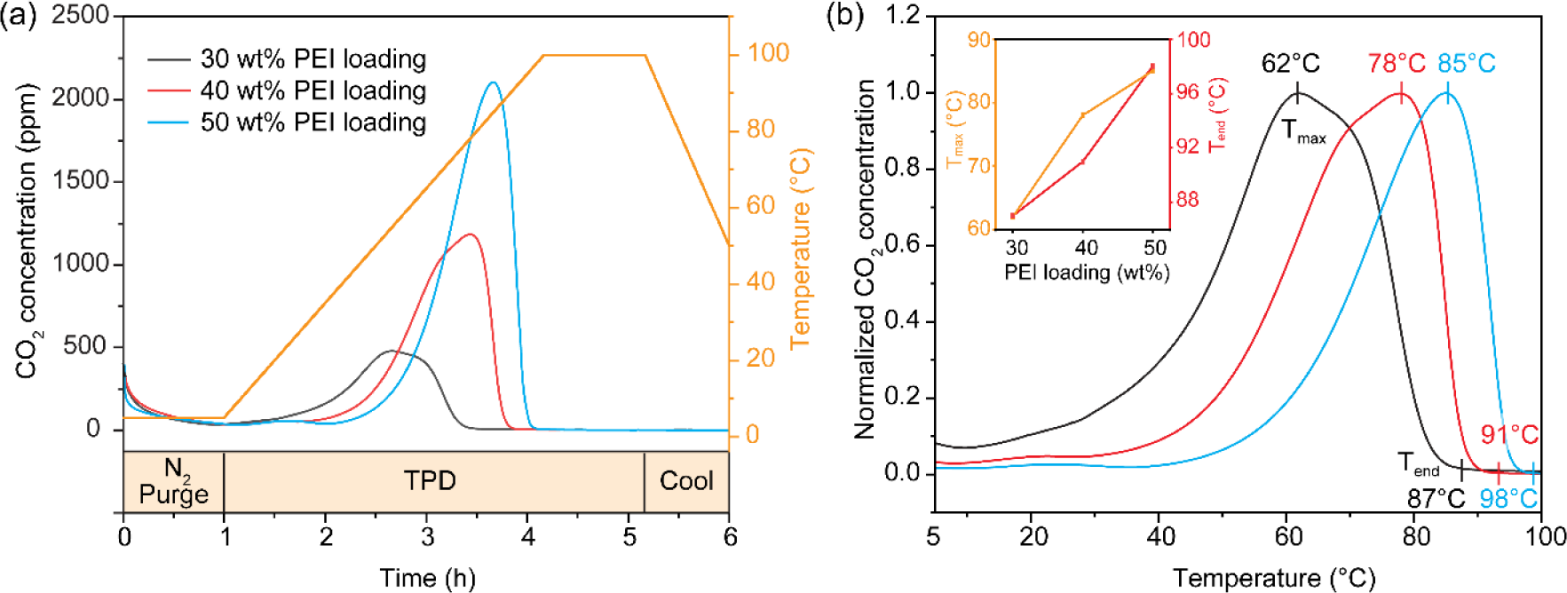
(a) CO_2_ TPD profiles of 30, 40, and 50 wt %-PEI/ePTFE/silica sorbents after adsorption at 5 °C and 80% RH. TPD was conducted under N_2_ flow (100 mL/min) using a fixed-bed system with the following steps: 1 h prepurging, temperature ramping at 0.5 °C/min to 100 °C, and 1 h isothermal hold at 100 °C, followed by cooling. (b) Normalized TPD profiles with *T*_max_ and *T*_end_ during temperature ramping step. The inset displays the variation of *T*_max_ and *T*_end_ depending on the PEI loading.

Therefore, if similar CO_2_ capacities can be achieved, sorbents with lower PEI loadings can provide a practical advantage by enabling faster CO_2_ desorption and regeneration at lower temperatures, ultimately improving the kinetics and reducing the energy cost for DAC operations. Although the present analysis is based on thermal N_2_ regeneration, practical DAC processes may involve steam-assisted regeneration. In these cases, the trade-off between hydrothermal stability and thermal energy demand must also be considered.

Furthermore, maintaining adsorbent stability during repeated adsorption–desorption cycles is crucial for ensuring consistent long-term performance. Our previous studies^25^ demonstrated the short-term cycling stability of PEI/ePTFE/silica sorbents under dry, wet, and steam regeneration conditions. Consistent with these findings, this study also confirms short-term cyclic stability of PEI/ePTFE/silica sorbents under both dry and humid adsorption conditions (Figure S21). While the present data confirm short-term cyclic stability, further studies will be needed to assess the long-term structural and oxidative stability of the PEI/ePTFE/silica system under realistic DAC conditions.

### Performance Evaluation Metrics: Mass vs Area Considerations for Sheet-Type Sorbents

Under most test conditions, the 40 wt %-PEI/ePTFE/silica showed the best balance between the CO_2_ capacity and sorption kinetics. However, it is worth considering whether the CO_2_ capacity expressed in mmol of CO_2_/g_sorbent_ is the most appropriate metric for evaluating adsorbent performance. For powdered or monolithic sorbents, mass-based productivity (mmol/g_sorbent_/h) is still a practical and relevant metric. However, for DAC systems using stackable sheet-type sorbents, the total sorbent usage is often limited by geometric area (length × width) rather than mass, so area-based productivity (mmol/inch^2^/h) can be a more appropriate normalization. When productivity was analyzed without considering regeneration time, the area-based results (Figure S22) differed from the mass-based trends (Figure S23). Specifically, the 30 wt %-PEI/ePTFE/silica showed the highest mass-based productivity during the first hour, but it was later surpassed by 40 and 50 wt %-PEI/ePTFE/silica (Figure 16). However, area-based productivity showed that the 40 wt %- and 50 wt %-PEI/ePTFE/silica performed well from the start, especially at 35 and 5 °C conditions. These findings highlight the importance of aligning performance evaluation metrics with the structural and operational design of the DAC system.

**Figure 16:**
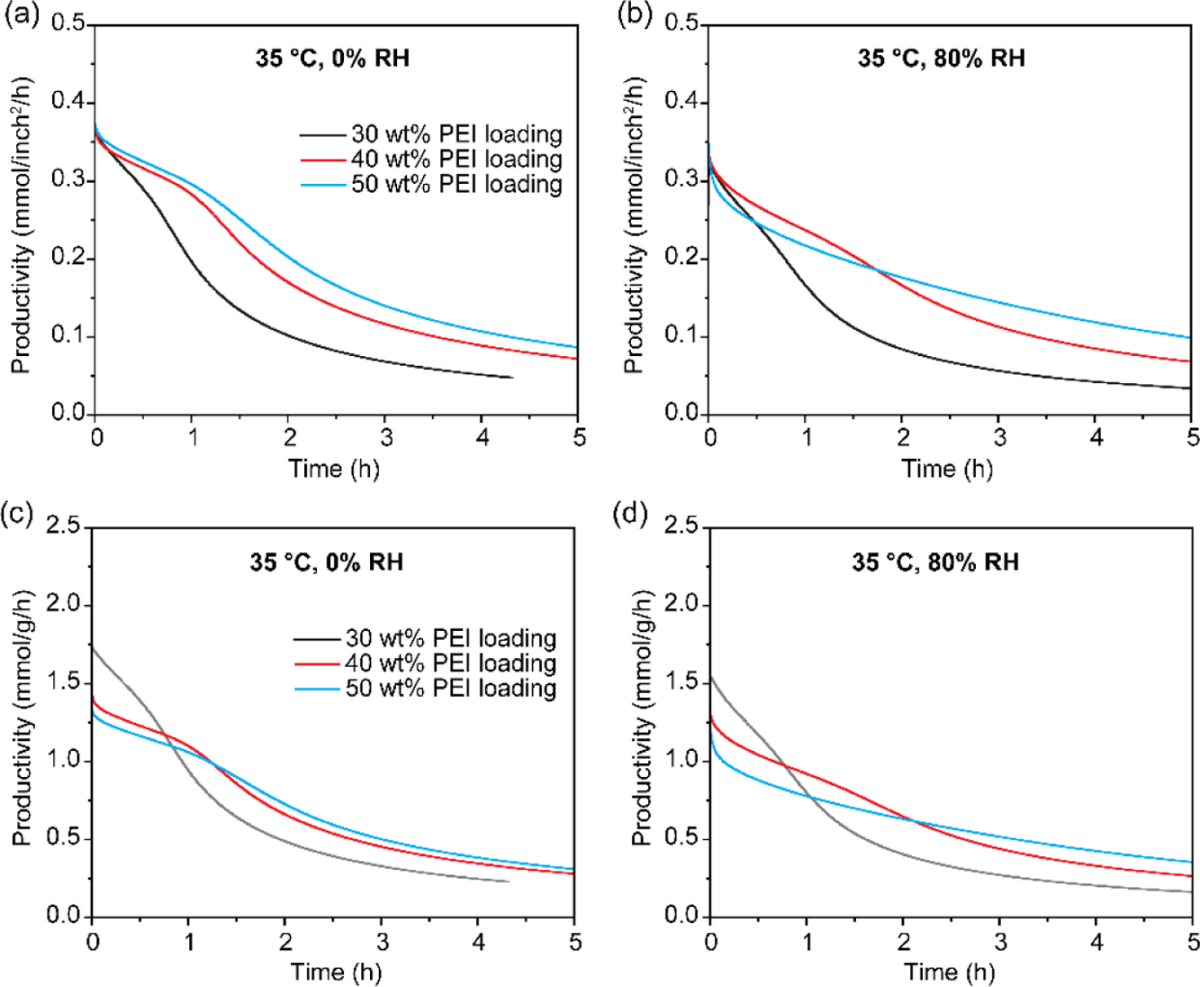
(a,b) Area-based CO_2_ productivity (mmol/inch^2^/h) and (c,d) mass-based CO_2_ productivity (mmol/g_sorbent_/h) of 30, 40, and 50 wt %-PEI/ePTFE/silica sorbents evaluated using a fixed-bed system at 35 °C under (a, c) dry (0% RH) and (b, d) humid (80% RH) conditions.

## Conclusions

This study systematically evaluated the DAC performance of PEI/ePTFE/silica sorbents in a fixed-bed system under simulated atmospheric conditions by varying the PEI-loading, temperature, and RH. Humidity significantly enhanced CO_2_ capacity at subambient temperature (−20 and 5 °C), but the effect was diminished at ambient temperature (35 °C). This is attributed to the improved PEI chain mobility and the promotion of carbamic acid conversion at lower temperatures. Although humidity provides a clear advantage for CO_2_ adsorption, water and CO_2_ compete for access to amine sites. This competitive effect is particularly pronounced at high absolute humidity (at 35 °C), where the moisture advantage is offset by site blocking. Across all conditions, the 50 wt %-PEI/ePTFE/silica generally exhibited the highest CO_2_ capacity, except in the low-temperature and low-humidity environments. However, the 50 wt %-PEI/ePTFE/silica showed a slower diffusion rate and a longer time to reach pseudoequilibrium due to kinetic limitations caused by the dense PEI network.

Therefore, optimizing the DAC system requires careful balance between capacity and kinetics. In the early stage of CO_2_ adsorption, the 30 wt %-PEI/ePTFE/silica outperformed the other samples at –20 °C, but the 40 wt %-PEI/ePTFE/silica became dominant over longer time periods. At 5 °C, the 40 wt %-PEI/ePTFE/silica maintained the best overall performance, but at 35 °C, the CO_2_ capacity gradually improved as the PEI loading increased from 40 to 50 wt %. These findings demonstrate that environmental conditions are the key factors in determining the optimal PEI loadings in achieving higher system performances. The results presented in this study aim to provide practical guidance for strategically selecting and optimizing amine-based sorbents for the intended climate and system configurations of a DAC system.

## Supplementary Material



## Notes

Additional experimental details including materials and methods, fixed-bed reactor setup, material characterization (SEM, TGA, and DSC), CO_2_ and H_2_O sorption isotherms, sorption capacity and kinetic data, and TPD results (PDF)
