# Supplementary material for: Climatic Conditions and Amine Loading Impact the Performance of Laminate-Supported Poly(ethylenimine) Direct Air Capture Sorbents
Source: JACS Au. 2025 Nov 25;5(12):6298–314. doi: 10.1021/jacsau.5c01294 (PMC12728659; doi:10.1021/jacsau.5c01294)
Supplement: Supplementary file 1 [file au5c01294_si_001.pdf]

Supporting Information

Climatic Conditions and Amine Loading Impact the Performance of Laminate-Supported Poly(ethylenimine) Direct Air Capture Sorbents

UnJin Ryu, Youn Ji Min, Wenyang Zhao, Yunseok Lee, Matthew J. Realff, Christopher W. Jones*

School of Chemical & Biomolecular Engineering, Georgia Institute of Technology, 311 Ferst Dr, Atlanta, Georgia 30332, United States

*Email: cjones@chbe.gatech.edu

Table of Contents

1. Materials and methods 3
1.1 Materials 3
1.2 Methods 3
Material characterizations. 3
Preparation of PEI/ePTFE/silica 5
2. Characterization of PEI/ePTFE/silica 6
3. DAC conditions 12
4. Fixed-bed experiments 14
4.1 Fixed-bed experiments setup under simulated atmospheric conditions 14
4.2 Fixed-bed experiments and related characterization 16

1. Materials and methods

1.1 Materials

Unless otherwise noted, all other reagents were obtained from commercial sources and used as received without purification. Poly(ethylenimine), branched (PEI, M_w~800 by LS, M_n~600 by GPC) purchased from Sigma-Aldrich. Methanol (MeOH, ACS grade, ≥99.8%) and hexane (ACS grade were ordered from VWR Chemicals BDH®. The customized ePTFE/silica was provided by W. L. Gore & Associates.

1.2 Methods

Material characterizations. The morphology of ePTFE/silica was evaluated using a field emission scanning electron microscope (FE-SEM, SU8230, Hitachi) and scanned by 1 kV. Pyrolysis thermogravimetric analysis (TGA) was performed using a TGA 550 (TA Instruments) under a nitrogen atmosphere from 100 to 700 °C at a heating rate of 10 °C/min with a flow rate of 90 mL/min after activation at 100 °C for 60 min. N_2 physisorption isotherms was measured using a TriStar II 3020 analyzer (Micromeritics Instrument Corporation), after activating the samples by evacuation at 90 °C for 6 h. The BJH pore size distribution was calculated from the desorption branch of the isotherm. Water vapor isotherms were collected at 5 °C, 20°C, 35 °C and 50 °C using a VSTAR water sorption analyzer (Anton Paar) after activating the sample under vacuum at 60 °C for 6 h. DSC analysis was performed using a DSC 250 (TA Instruments) under a nitrogen atmosphere with a heating rate of 10 °C/min from –80 °C to 50 °C. Each sample (two 4 mm discs with identical area) was pre-activated under vacuum at 60 °C for 1 h in the DSC pan to remove sorbed moisture and CO_2. After activation, the sample was moved to a chamber connected to a gas

purging system and exposed to the designated gas stream at room temperature (27 °C) under dry CO_2 (400 ppm), humid N_2, or humid CO_2. The humid gas streams were generated using a water humidifier connected to the inlet of the test chamber. Adsorption and equilibration were conducted until the CO_2 concentration at the chamber outlet became constant, as confirmed by an IR detector (LI-850, LiCOR). The equilibrated samples were then immediately sealed in hermetic pans to ensure a closed system during measurement. Dry CO_2 uptake for baseline assessment was measured using a TGA Q500 (TA Instruments). Each sample was punched into a uniform 5 mm diameter disk using a circular punch and was first activated under nitrogen at 100 °C for 2 h, cooled to 35 °C under nitrogen. The activated sample was then exposed to a 400 ppm dry CO_2 /N_2 flow (90 mL/min), and the uptake was measured for 6 h until equilibrium was reached. *In situ* diffuse reflectance infrared Fourier transform spectroscopy (DRIFTS) was performed using a Nicolet iS10 IR spectrometer equipped with a low-temperature DRIFTS cell (CHC-CHA-4, Harrick Scientific). A 6 mm-diameter punched sample was loaded into the cell and activated at 100 °C under N_2 flow (50 mL/min) for 1 h. After cooling to the target adsorption temperature (-20°C, 5°C or 35°C), the gas stream was switched to experimental condition. For dry experiments, dry 400 ppm CO_2/N_2 (50 mL/min) was introduced. For humid experiments, wet N_2 (50 mL/min) was flowed until the outlet water concentration stabilized at the target humidity level (confirmed using a CO_2/H_2O analyzer, LI-COR 850, Bioscience), followed by switching to humidified 400 ppm CO_2/N_2 (50 mL/min). The humidified gas was generated using potassium carbonate[1] based bubbler system and diluted the humidity with dry N_2 to achieve the desired relative humidity. IR spectra were collected every 2 min with 128 scans at 8 cm^{-1} resolution.

Preparation of PEI/ePTFE/silica. This preparation was adapted from a previously reported procedure.[2] The bare ePTFE/silica materials cut into desired sizes such as 0.5”×1” or 0.5”×2” and were activated under vacuum at 100 °C overnight. The activated sheets were first immersed in pure MeOH to pre-wet the material and eliminate trapped air bubbles. Subsequently, the wet sheets were transferred into PEI/MeOH solutions and left them at room temperature for 4 h without stirring. The PEI concentrations of PEI/MeOH are listed in **Table 1** of main manuscript. The methanol volume was adjusted based on a fixed ratio of 10 mL per 100 mg of ePTFE/silica sheet. After impregnation, ePTFE/silica sheets were soaked in pure hexane for1 h without stirring. Finally, the materials were air-dried overnight and further dried under vacuum at 60 °C for 6 hours.

2. Characterization of PEI/ePTFE/silica

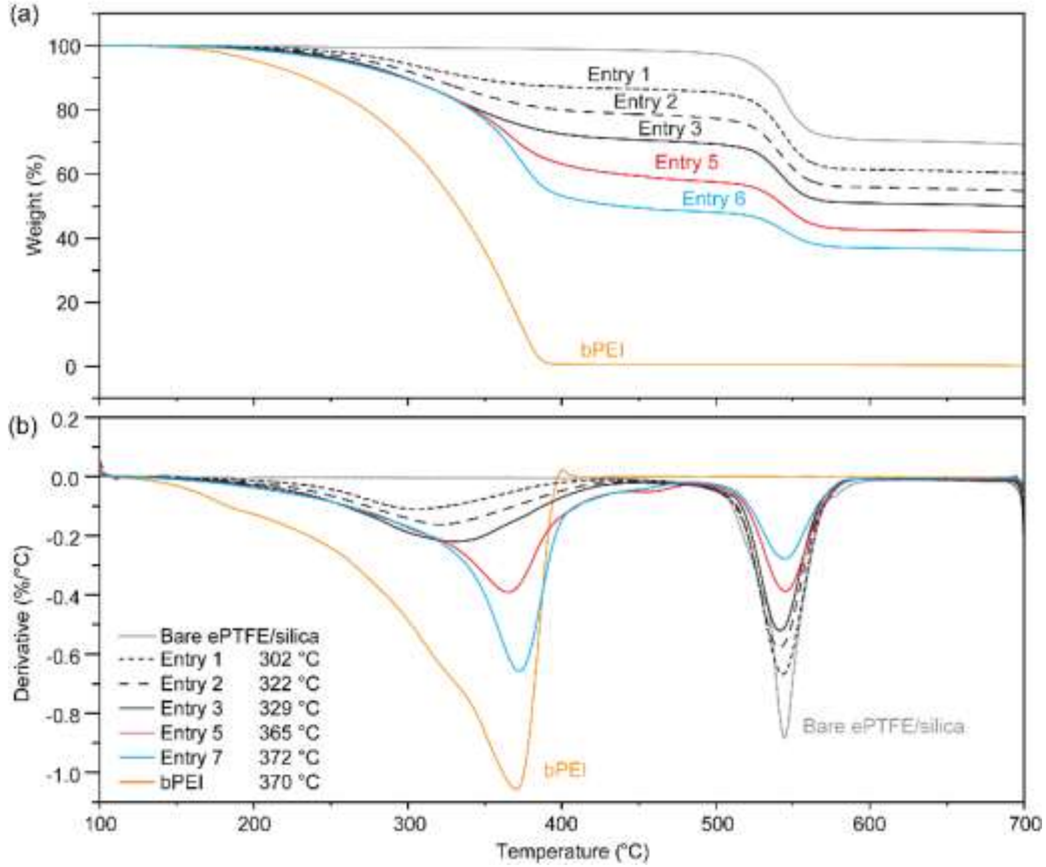

Figure S1. Comparative (a) Pyrolysis TGA curves and (b) DTG curves and T_{max} values of bPEI (Entry 1, Entry 2, Entry 3, Entry 5, Entry 7 and bPEI). The Entry numbers correspond to those listed in **Table 1** of the main manuscript.

Table S1. PEI loading by mass vs. TGA. The Entry numbers correspond to those listed in **Table 1** of the main manuscript.

Entry	PEI loading by mass (wt%)	PEI loading by TGA (wt%)
1	13.2	13.6
2	20.5	21.6
3	27.0	29.6
5	38.3	42.2
7	49.1	51.5

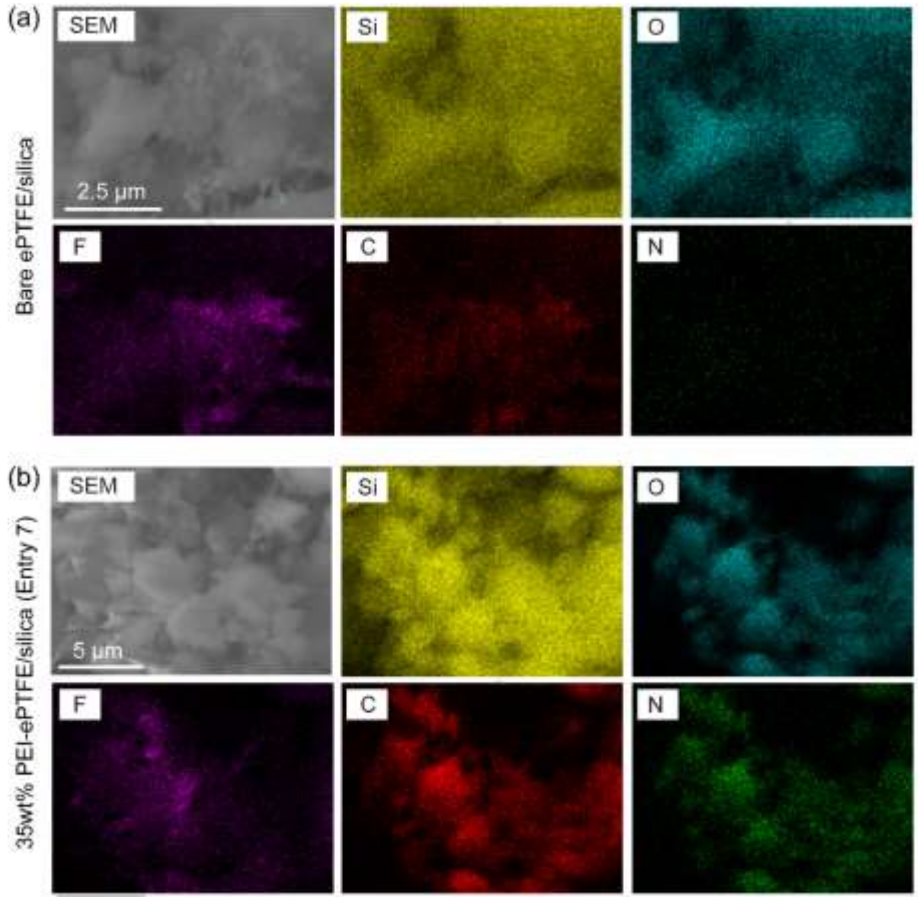

Figure S2. SEM images and corresponding EDS elemental maps showing the distribution of Si and O (from silica), F and C (from ePTFE), and C and N (from branched PEI) (a) Bare ePTFE/silica and (b) 35wt% PEI-ePTFE/silica (Entry 7). The Entry numbers correspond to those listed in **Table 1** of the main manuscript.

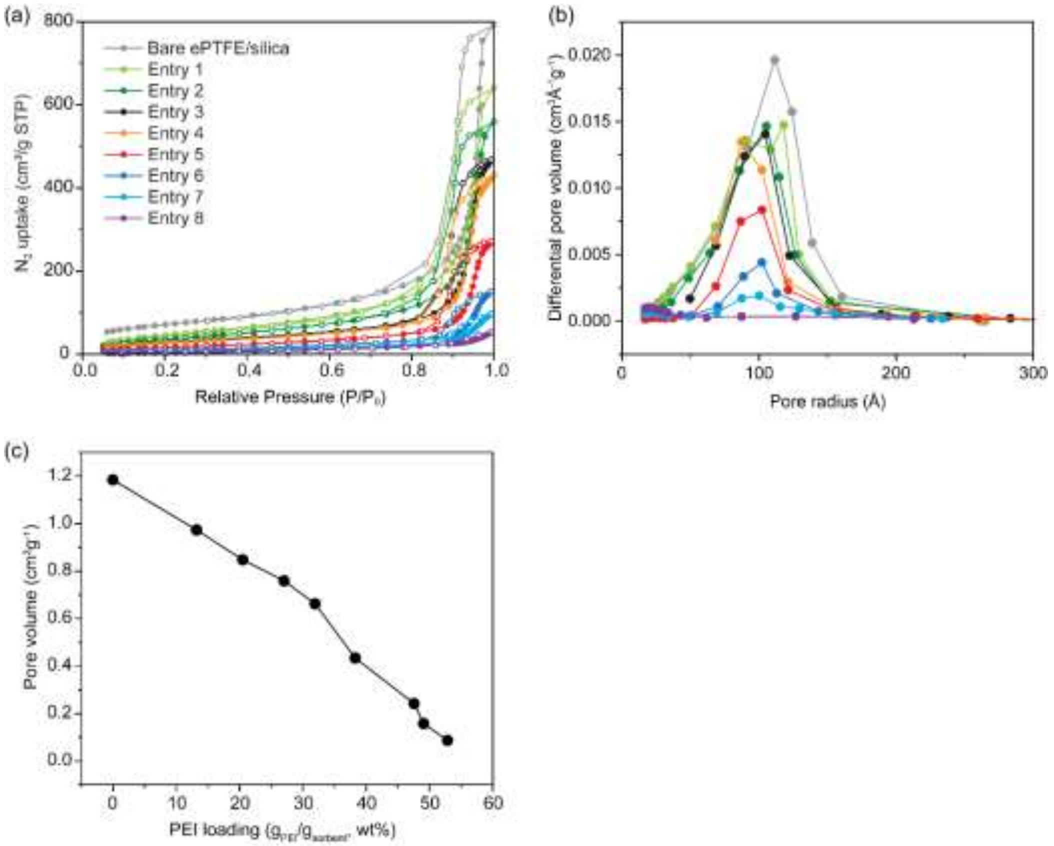

Figure S3. (a) N_2 physisorption isotherms at 77 K, (b) corresponding pore size distributions, and (c) pore volumes of ePTFE/silica sorbents with varying PEI loadings. The Entry numbers correspond to those listed in **Table 1** of the main manuscript.

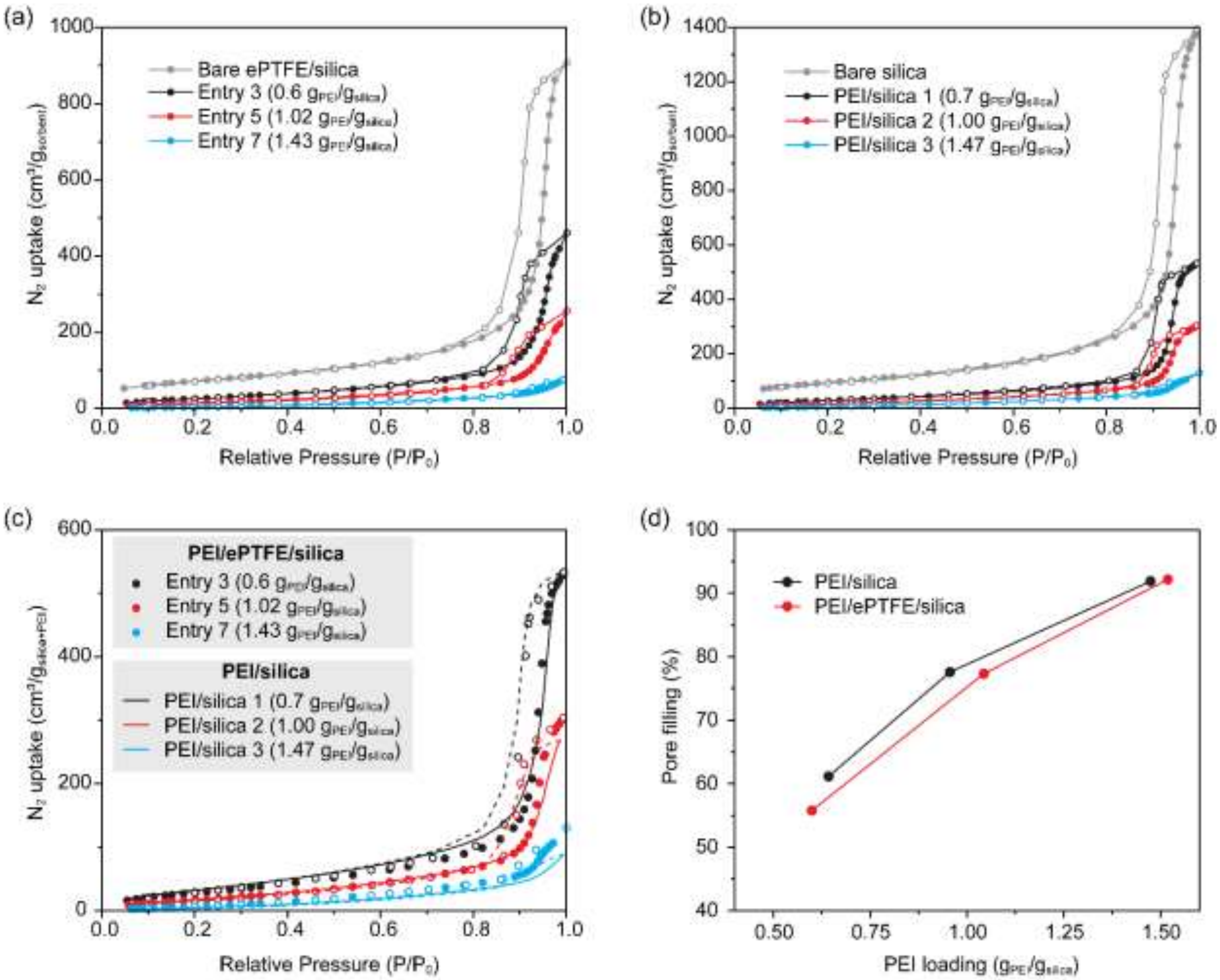

Figure S4. N_2 isotherm of (a) PEI/ePTFE/silica, (b) PEI/silica, both at 77 K. Here, $g_{sorbent}$ includes the total mass of silica, PEI and ePTFE. Comparison of N_2 isotherms of (c) PEI/ePTFE/silica and PEI/silica based on the combined mass of silica and PEI, excluding ePTFE. (d) Pore filling (%) of PEI/ePTFE/silica and PEI/silica.

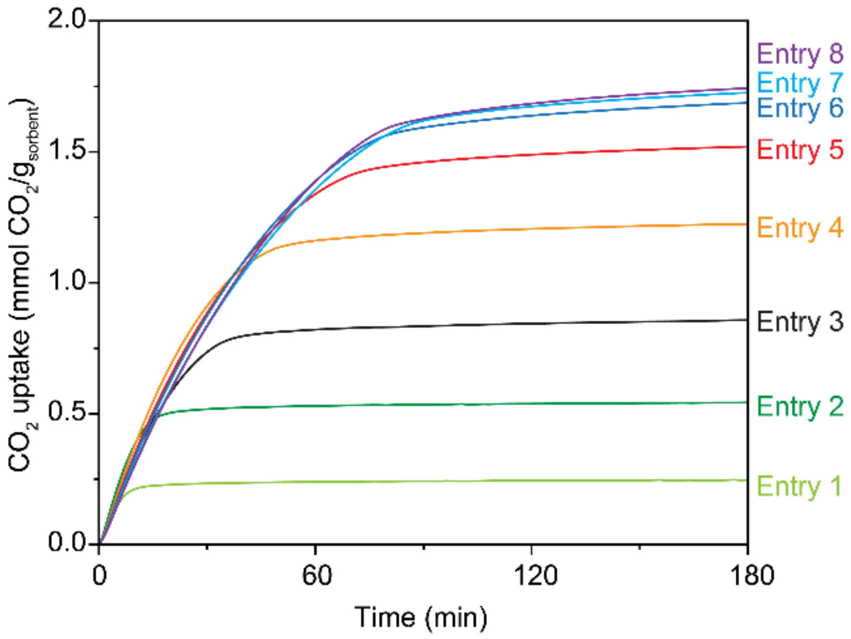

Figure S5. Absolute CO_2 uptake profiles (in mmol $CO_2/g_{sorbent}$) of PEI/ePTFE/silica sorbents from Entry 1 (13.2 wt%-PEI loading) to Entry 7 (52.9 wt%-PEI loading) at 35°C under dry conditions using a TGA. The Entry numbers correspond to those listed in **Table 1** of the main manuscript. Here, $g_{sorbent}$ includes the total mass of silica, PEI and ePTFE. Each material (5 mm diameter round) had the following average masses (± SD, mg): Entry 1 = 5.73 ± 0.19, Entry 2 = 6.19 ± 0.52, Entry 3 = 7.05 ± 0.05, Entry 4 = 7.50 ± 0.22, Entry 5 = 8.40 ± 0.33, Entry 6 = 9.00 ± 0.59, Entry 7 = 9.57 ± 0.08, and Entry 8 = 10.51 ± 0.32.

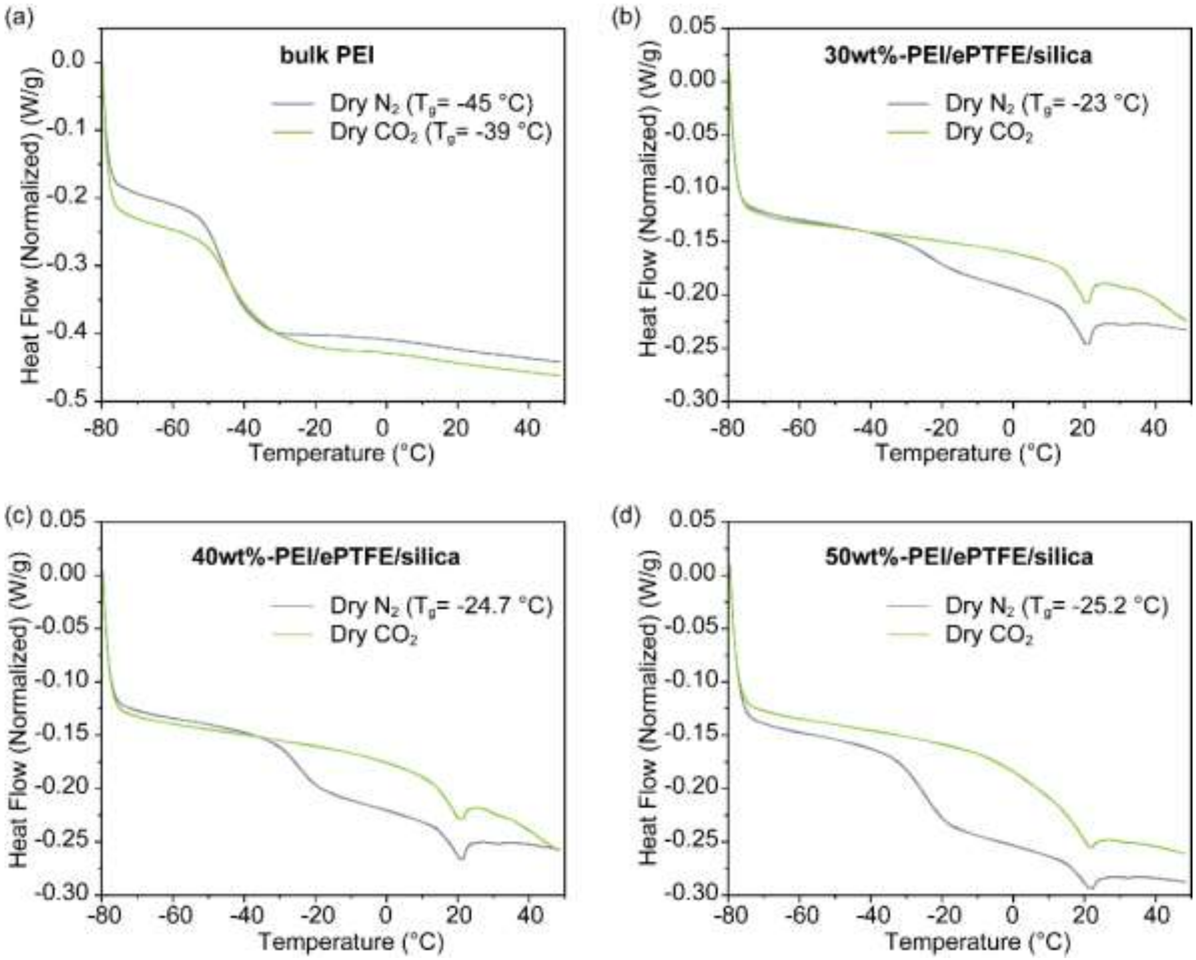

Figure S6. DSC thermograms of (a) bulk PEI, (b) 30 wt%-PEI/ePTFE/silica, (c) 40 wt%-PEI/ePTFE/silica and (d) 50 wt%-PEI/ePTFE/silica. Samples were pre-activated under vacuum at 60 °C overnight. For the "Dry N_2" condition (gray), samples were exposed the to dry N_2. For the "Dry CO_2" condition (green), the same samples were subsequently exposed to 400 ppm CO_2/N_2.

3. Simulated atmospheric conditions for DAC

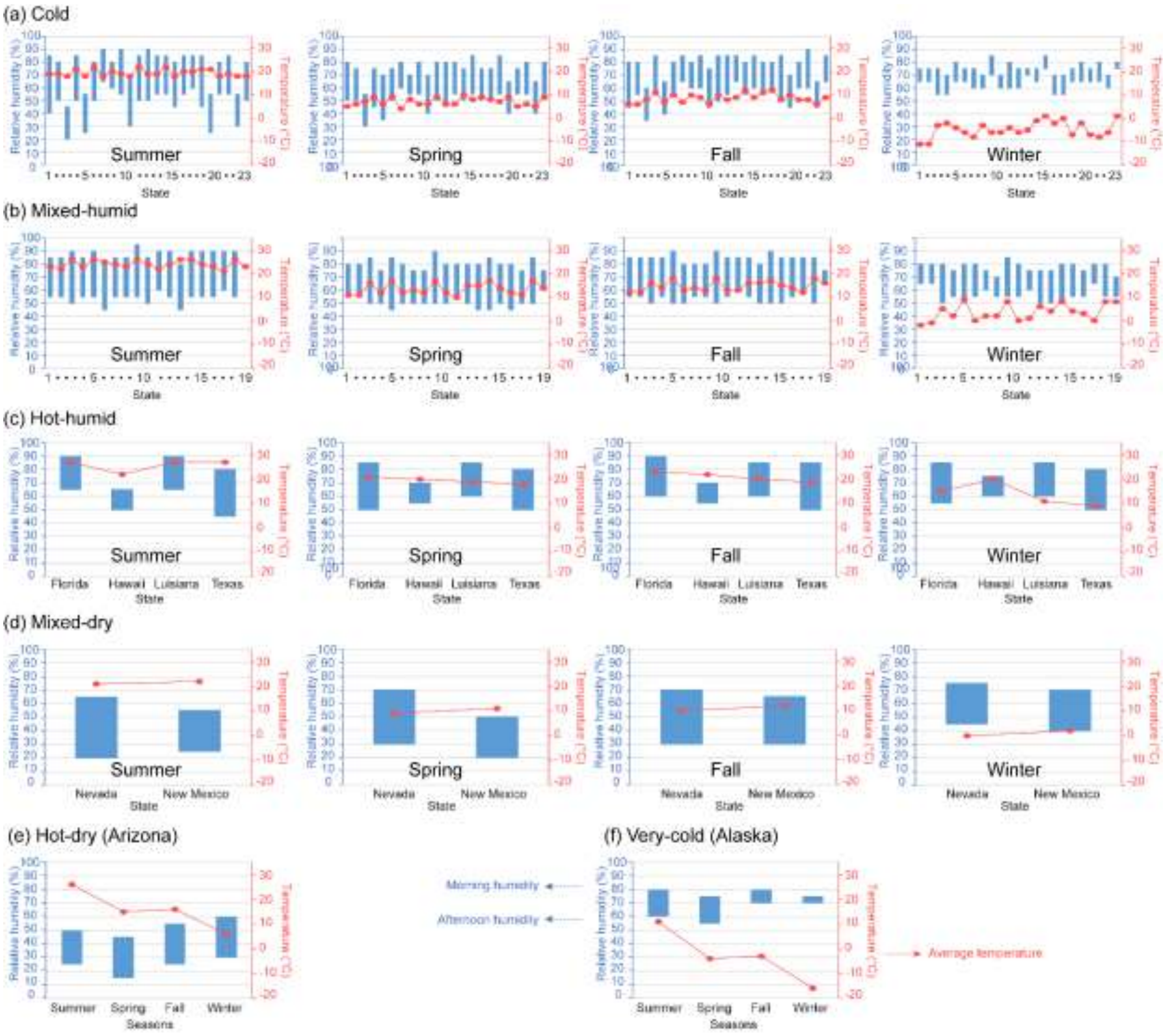

Figure S7. American climate zones and average annual temperature for each US state. The average temperatures and humidities are based on data collected by weather stations throughout each state during the years 1971 to 2000 and made available by the NOAA National Climatic Data Center of the United States.[3] The climate zones were classified based on the *Guide to Determining Climate Zones by County* report.[4] For each state, the figure provides average seasonal (spring, summer, fall, winter) morning and afternoon relative humidity, as well as average seasonal temperatures. (a) Cold zone (State 1: North Dakota, 2: Minnesota, 3: Colorado, 4: Connecticut, 5: Idaho, 6: Iowa, 7: Maine, 8: Massachusetts, 9: Michigan, 10: Montana, 11: Nebraska, 12: New Hampshire, 13: New York, 14: Ohio, 15: Oregon, 16: Pennsylvania, 17: Rhode Island, 18: South Dakota, 19: Utah, 20: Vermont, 21: Wisconsin, 22: Wyoming, 23:Washington), (b) mixed-humid zone (State 1: Illinois, 2: Indiana, 3: Arkansas, 4: Delaware,5: Georgia, 6: Kansas, 7: Kentucky, 8: Maryland, 9: Mississippi, 10: Missouri, 11: New Jersey, 12: North Carolina, 13: Oklahoma, 14: South Carolina, 15: Tennessee, 16: Virginia, 17: West Virginia, 18: Alabama, 19: California) (c) hot-humid zone (State: Florida, Hawaii, Luisiana, Texas), (d) mixed-dry zone (Nevada, New Mexico), (e) hot-dry zone (Arizona) and (f) very-cold zone (Alaska).

Table S2. Seasonal average temperature and relative humidity by U.S. climate zone. Data represent averages from 1971–2000 NOAA datasets. Morning and afternoon relative humidity (RH) values are reported as averages across all states within each zone. This table was prepared based on **Figure S7**.

Climate Zone	Temperature/Humidity	Summer	Spring	Fall	Winter
Very Cold	Average temperature (°C)	11	−4	−3	−16
	Average morning RH (%)	80	75	80	75
	Average afternoon RH (%)	60	55	70	70
Cold	Average temperature (°C)	19	7	9	−5
	Average morning RH (%)	85	75	80	75
	Average afternoon RH (%)	50	50	55	65
Mixed-Humid	Average temperature (°C)	24	13	14	3
	Average morning RH (%)	90	80	85	80
	Average afternoon RH (%)	55	50	55	55
Hot-Humid	Average temperature (°C)	27	19.5	21	13
	Average morning RH (%)	85	82.5	85	82.5
	Average afternoon RH (%)	57.5	52.5	57.5	57.5
Mixed-Dry	Average temperature (°C)	21.5	10	11	1
	Average morning RH (%)	60	60	67.5	72.5
	Average afternoon RH (%)	22.5	25	30	42.5
Hot-Dry	Average temperature (°C)	26	15	16	6
	Average morning RH (%)	50	45	55	60
	Average afternoon RH (%)	25	15	25	30

4. Fixed-bed experiments

4.1 Fixed-bed experiments setup under simulated atmospheric conditions

Fixed-bed adsorption experiments were conducted under controlled temperature and humidity conditions to simulate atmospheric environments. Temperature was regulated using a bench-top temperature chamber (SH-642, ESPEC) capable of providing temperatures from −40°C to 160°C. Humidity at each temperature was controlled by a dew point generator (LI-610, LiCOR). The CO_2 and H_2O concentrations in the gas stream were continuously monitored using an IR gas analyzer (LI-850, LiCOR). The test reactor consisted of a cylindrical stainless-steel body with a 3D-printed plastic insert to minimize gas bypass. Each sample was cut into 0.5"×1" rectangular sheets and mounted vertically in the plastic holder. The typical mass of the single-sheet sorbent samples was 0.102 ±0.003 g for 30 wt%-PEI/ePTFE/silica, 0.128±0.005 g for 40 wt%-PEI/ePTFE/silica and 0.139±0.001 g for 50 wt%-PEI/ePTFE/silica. The sheet was sandwiched between mesh nets to hold them securely and ensure uniform gas contact, minimizing bypass along the reactor wall (**Figure S8**). To isolate material performance and avoid lamination effects, single-sheet samples were used. Gas flow rates of 100 mL/min or 200 mL/min were applied during pre-saturation and CO_2 adsorption steps, depending on the specific test conditions. Temperature-programmed desorption (TPD) data were also collected immediately after adsorption using pre-set heating profiles in the bench-top temperature chamber.

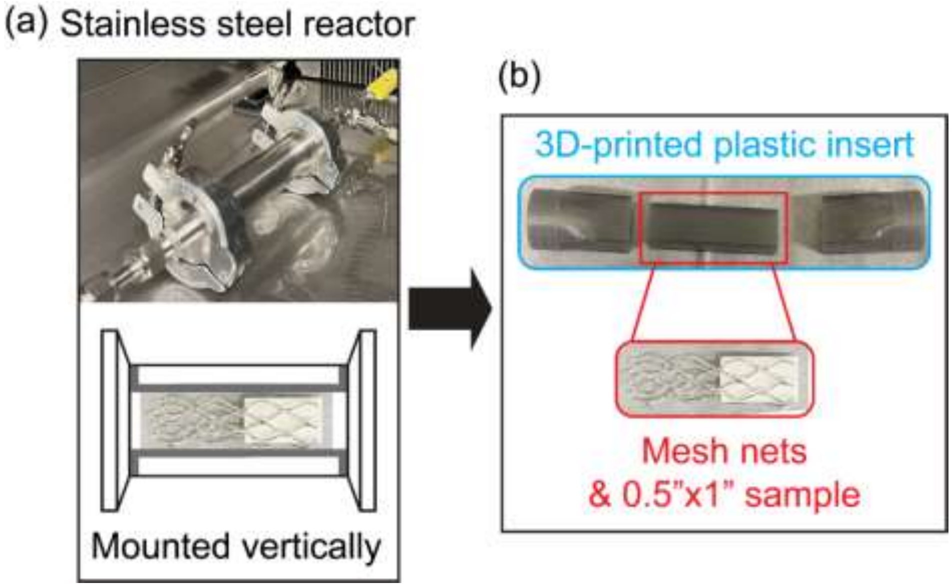

Figure S8. Schematic and photograph of the fixed-bed reactor setup used for CO_2 adsorption experiments. (a) The stainless-steel reactor houses a 3D-printed plastic insert. (b) A single 0.5″×1″ sample is mounted vertically between mesh nets and placed within the holder.

4.2 Fixed-bed experiments and related characterization

Table S3. CO_2 capacity at different temperatures and different relative humidities for 30 wt%-PEI/ePTFE/silica, 40 wt%-PEI/ePTFE/silica and 50 wt%-PEI/ePTFE/silica using a fixed-bed system.

Sample (PEI loading)	Temperature (°C) / RH (%)	−20	5	35
30 wt%	0	0.74	0.83	0.99
	20	**0.41****	1.15	1.07
	50	1.64	1.47	1.01
	80	**1.77***	1.56	0.81
40 wt%	0	**0.61****	0.92	1.45
	20	0.99	1.74	1.70
	50	2.57	2.54	1.57
	80	**2.61***	2.50	1.33
50 wt%	0	**0.45****	0.83	1.62
	20	0.57	1.61	2.09
	50	2.31	3.38	2.05
	80	**3.49***	2.89	1.84

* Maximum CO_2 capacity value for each sample. ** Minimum CO_2 capacity value for each sample.

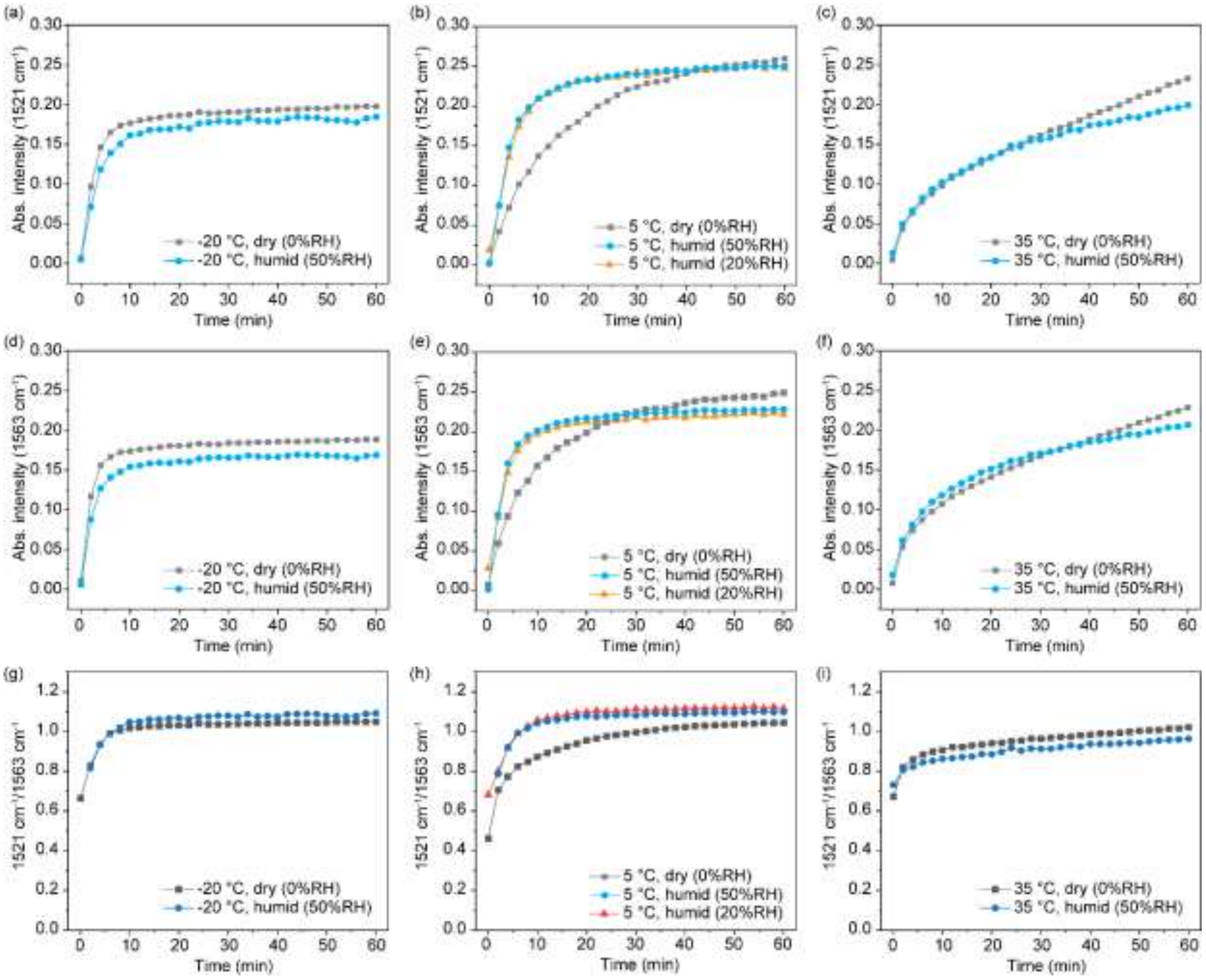

Figure S9. IR intensity profiles of (a–c) NH_3^+/NH_2^+ deformation (1521 cm^{-1}), (d–f) carbamate (1563 cm^{-1}) and (g–i) 1521 cm^{-1}/1563 cm^{-1} absorbance ratio for 40 wt%-PEI/ePTFE/silica during 1h of CO_2 exposure under dry and humid conditions at various temperatures. For the 5 °C case (b, e, h), additional data collected at 20 % RH are included for comparison with the 50 % RH results.

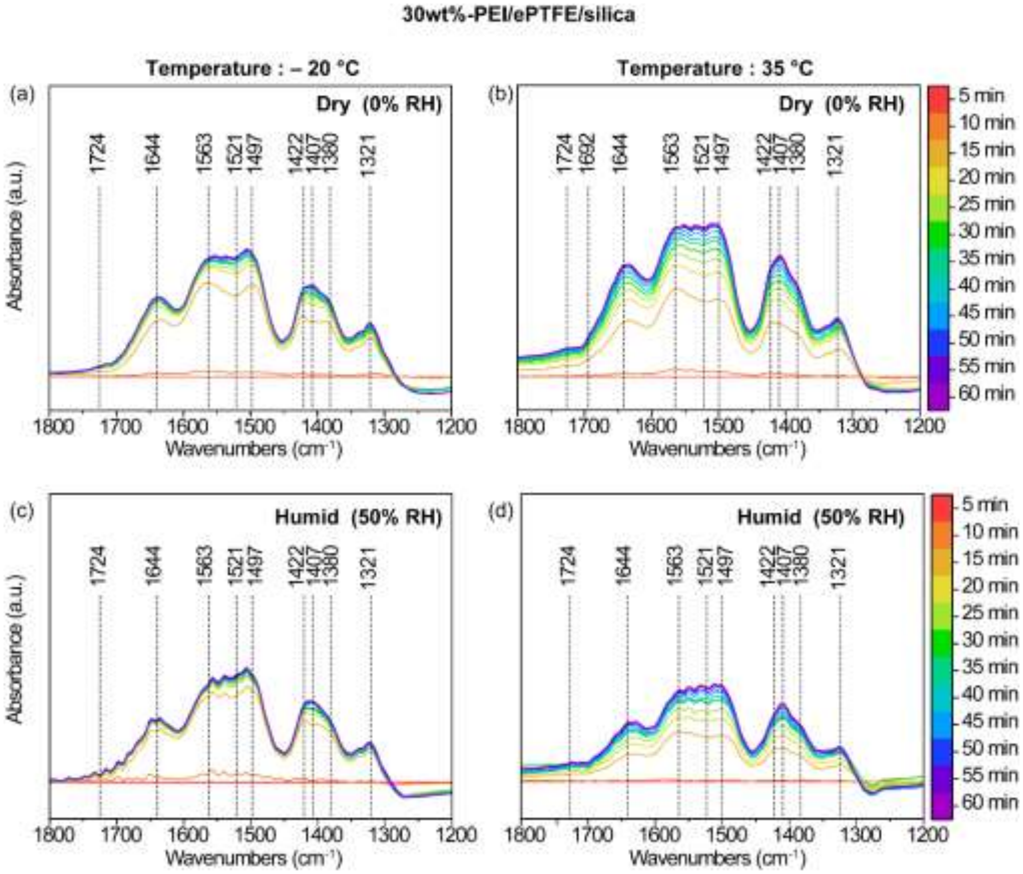

Figure S10. *In situ* IR spectra of 30 wt%-PEI/ePTFE/silica during CO_2 exposure for 1 hour under dry and humid conditions at various temperatures. (a–b) show spectra under dry conditions at (a) -20 °C and (b) 35 °C. (c–d) show spectra under 50% RH conditions at (c) −20 °C and (d) 35 °C.

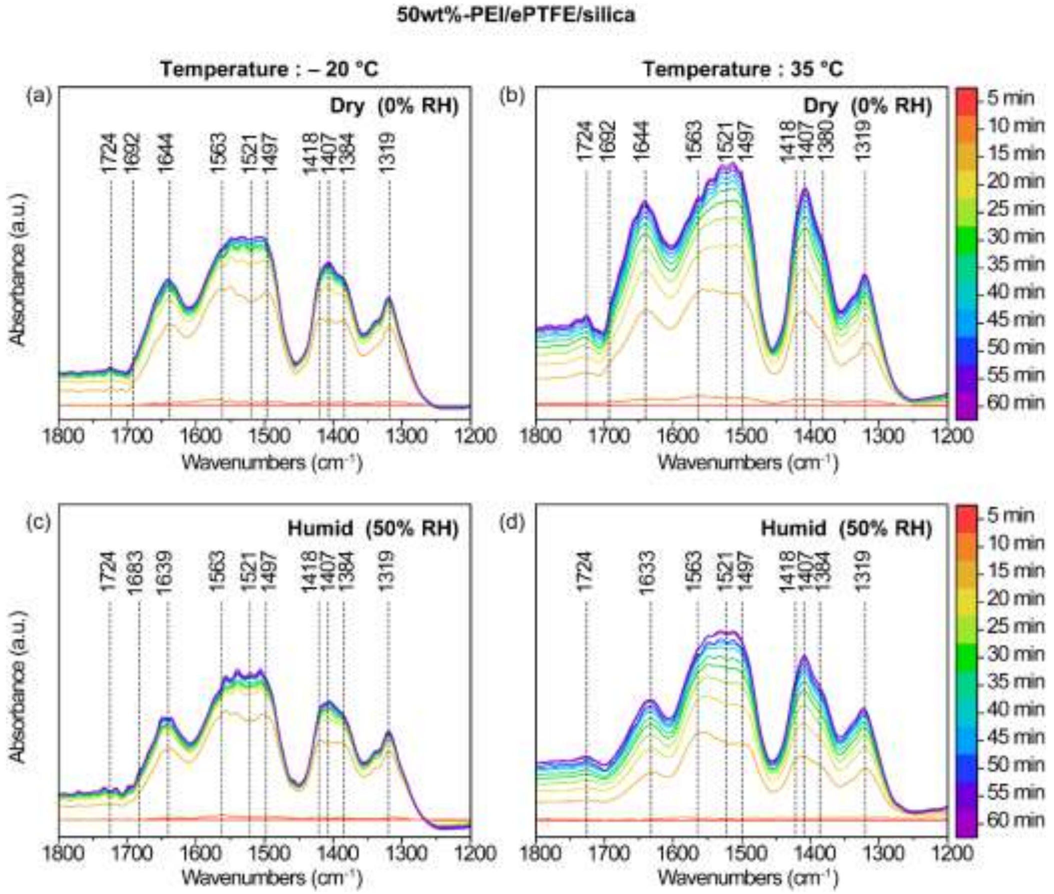

Figure S11. *In situ* IR spectra of 50 wt%-PEI/ePTFE/silica during CO_2 exposure for 1 hour under dry and humid conditions at various temperatures. (a–b) show spectra under dry conditions at (a) −20 °C and (b) 35 °C. (c–d) show spectra under 50% RH conditions at (c) −20 °C and (d) 35 °C.

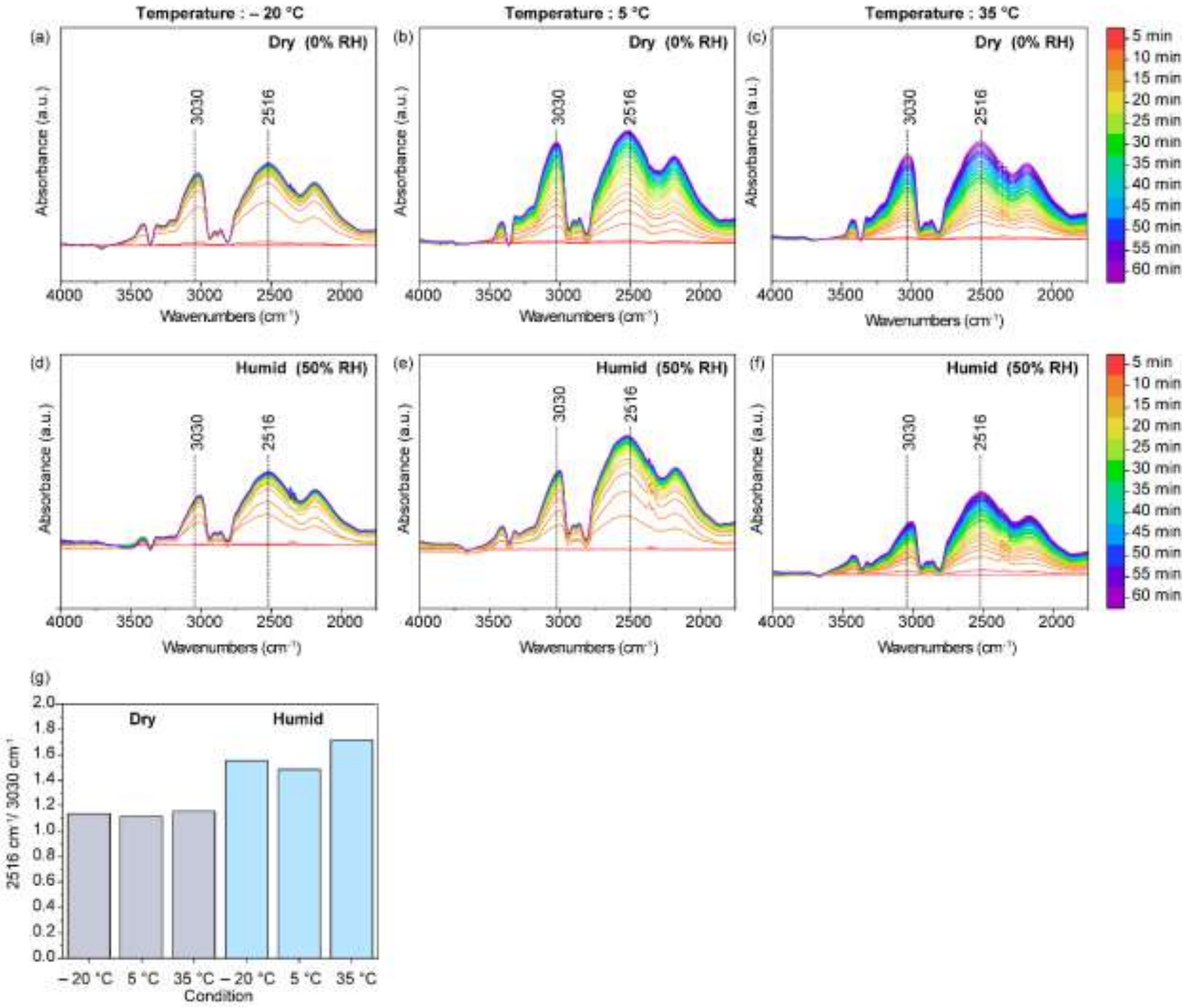

Figure S12. *In situ* IR spectra of 40 wt%-PEI/ePTFE/silica during CO_2 exposure for 1 hour under dry and humid conditions at various temperatures. (a–c) show spectra under dry conditions at (a) -20 °C, (b) 5 °C and (c) 35 °C. (d–f) show spectra under 50% RH conditions at (d) −20 °C, (e) 5 °C and (f) 35 °C. (g) 2516 cm^{-1}/3030 cm^{-1} absorbance ratio under dry and humid conditions.

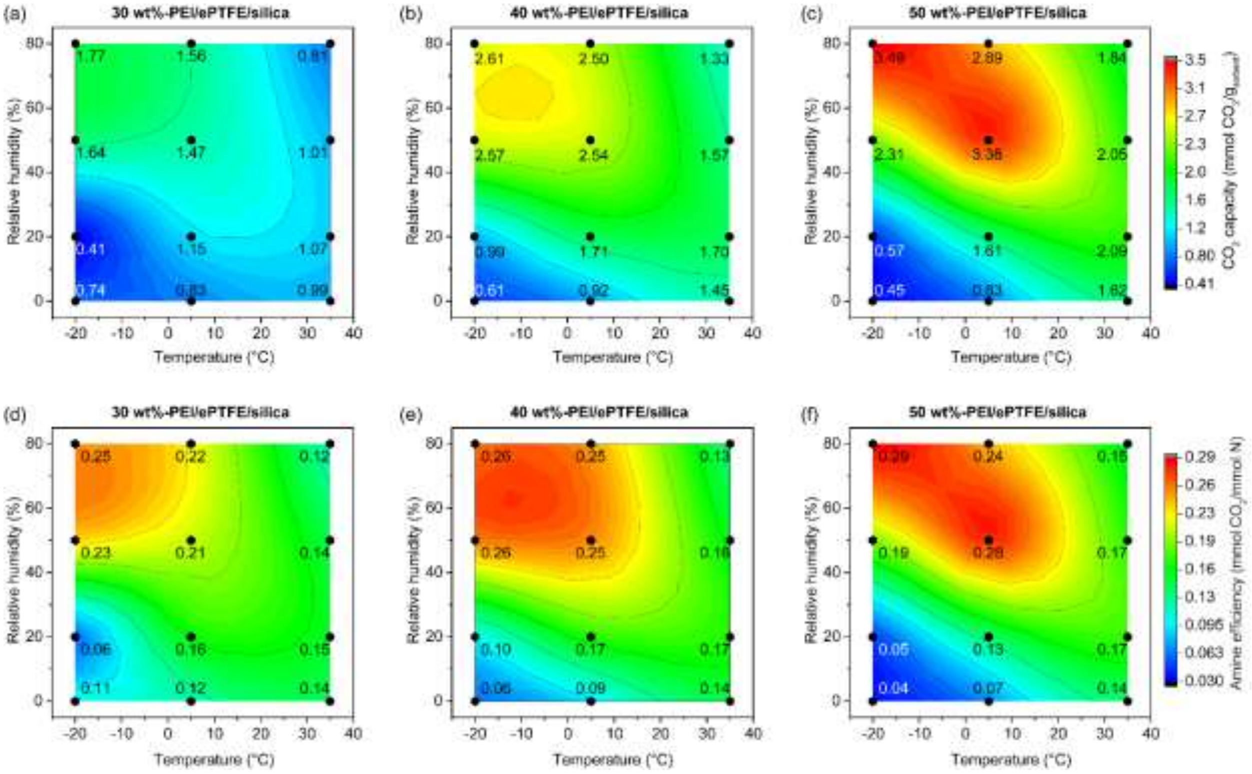

Figure S13. (a–c) Contour map of CO_2 capacity at different temperature and different relative humidity for (a) 30 wt%-PEI/ePTFE/silica (b) 40 wt%-PEI/ePTFE/silica and (c) 50 wt%-PEI/ePTFE/silica using fixed-bed system. All contour maps were plotted using the same capacity range (0.41–3.5 mmol/g). (d–f) Contour map of amine efficiency at different temperature and different relative humidity for (d) 30 wt%-PEI/ePTFE/silica (e) 40 wt%-PEI/ePTFE/silica and (f) 50 wt%-PEI/ePTFE/silica. All contour maps were plotted using the same capacity range (0.03–0.29 mmol/g).

Table S4. Water concentration in humid gas (ppm)

Temperature	0% RH	20% RH	50% RH	80% RH
35	0	11461	29153	47475
5	0	1762	4416	7085
−20	0	253	633	1013

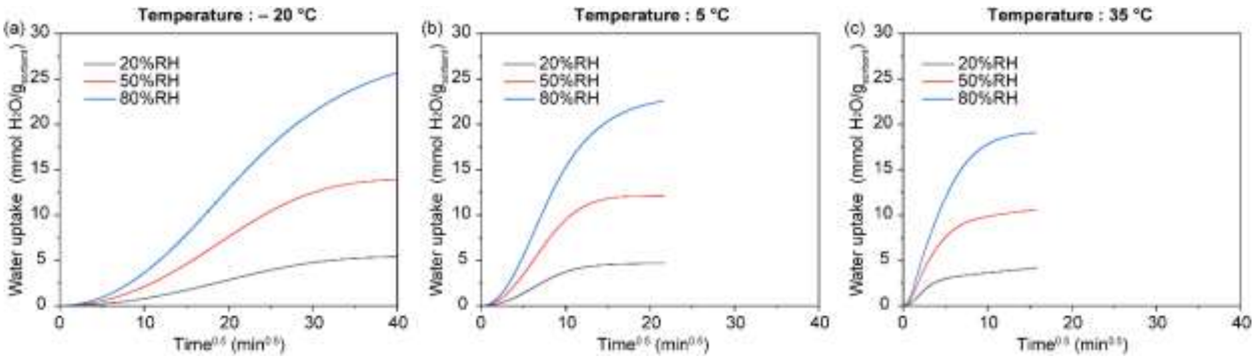

Figure S14. Absolute water uptake profiles (in mmol $H_2O/g_{sorbent}$) of 40 wt%-PEI/ePTFE/silica sorbents at (a) −20°C, (b) 5°C and (c) 35°C under various relative humidities using fixed-bed system. (The data set was truncated at 40, 21 and 16 min respectively after the start of the experiment to highlight the variations in the uptake slope under different temperature)

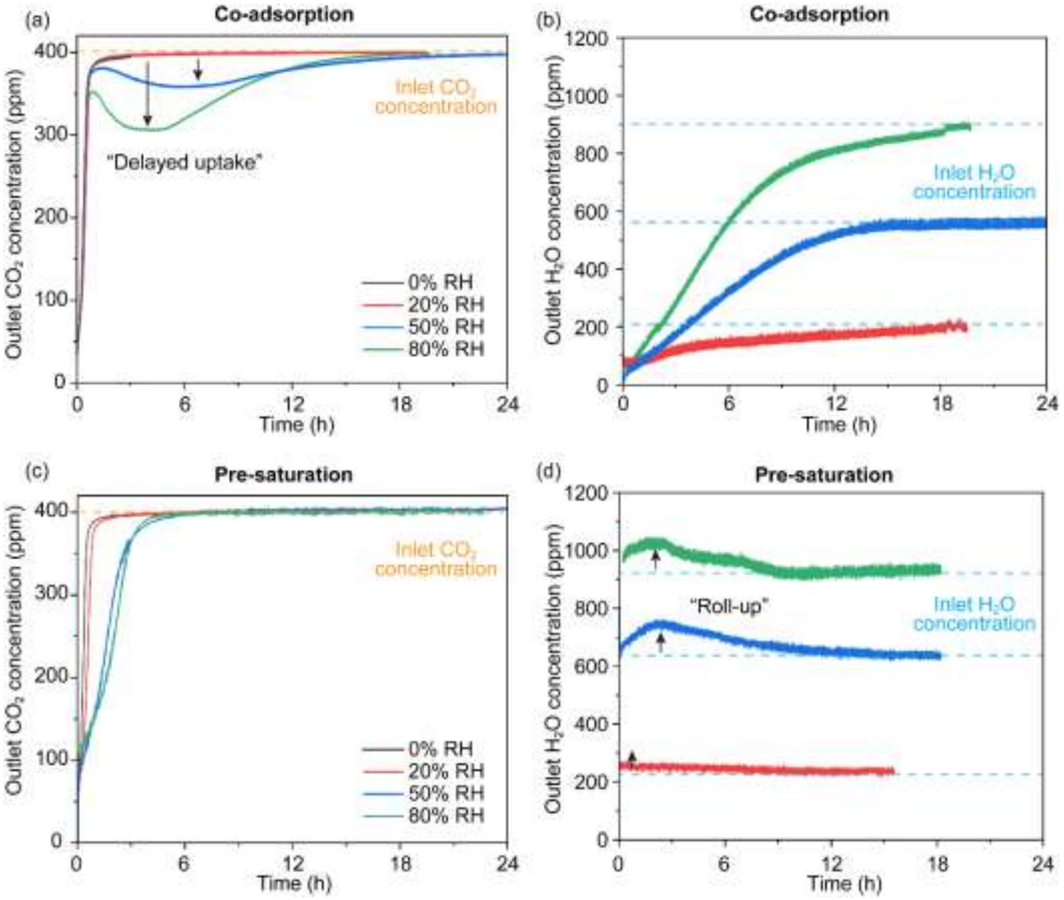

Figure S15. CO_2 and H_2O uptake profiles at –20 °C using 40 wt%-PEI/ePTFE/silica under different humidity conditions. (a,b) Co-adsorption method, where CO_2/N_2 and H_2O are introduced simultaneously. (c,d) Pre-saturation method, where the sorbent is first saturated with H_2O/N_2 followed by CO_2/N_2 introduction.

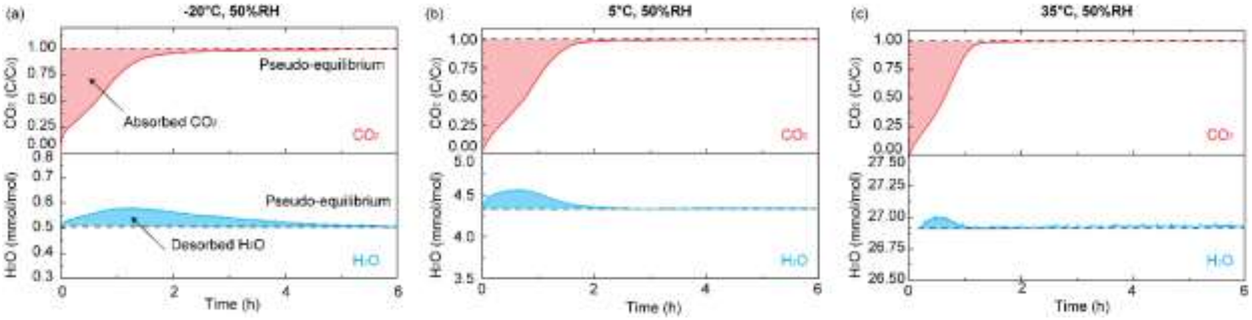

Figure S16. Representative normalized CO_2 uptake profiles (C/C_0) and corresponding water desorption profiles (mmol/mol) during the humid CO_2 sorption step at (a) −20°C, (b) 5 °C and (c) 35 °C under 50% RH using the 30 wt% PEI/ePTFE/silica sorbent.

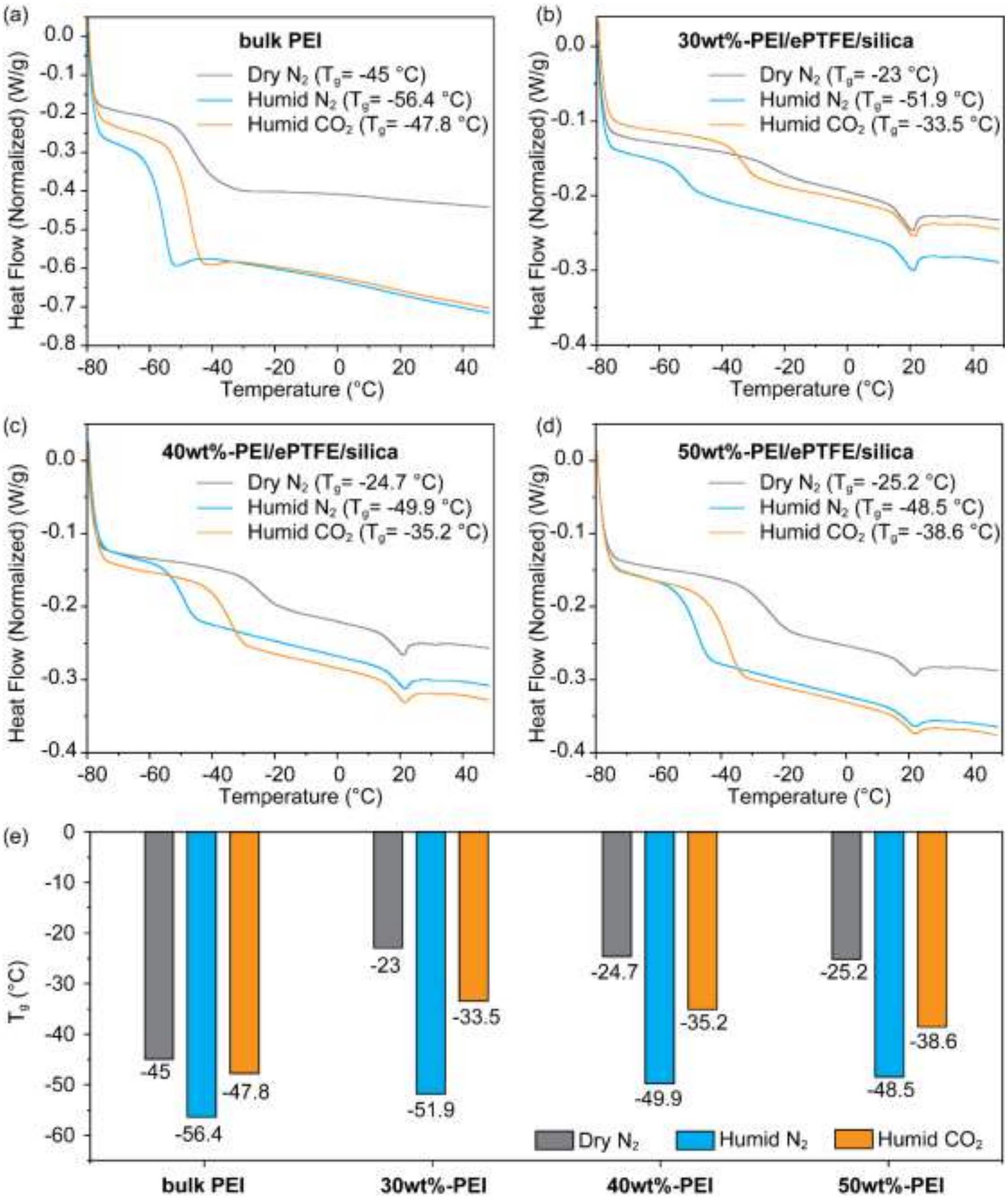

Figure S17. DSC thermograms of (a) bulk PEI, (b) 30 wt%-PEI/ePTFE/silica, (c) 40 wt%-PEI/ePTFE/silica and (d) 50 wt%-PEI/ePTFE/silica. Samples were pre-activated under vacuum at 60 °C overnight. For the "Dry N_2" condition (gray), samples were exposed the to dry N_2. For the "Humid N_2" condition (blue), the same samples were subsequently exposed to H_2O/N_2 with 50% RH at 27 °C (room temperature). For the "Humid CO_2" condition (orange), the same samples were subsequently exposed to 400 ppm CO_2/N_2 with 50% RH at 27 °C (room temperature). (e) Summary of glass transition temperatures (Tg) extracted from (a–d).

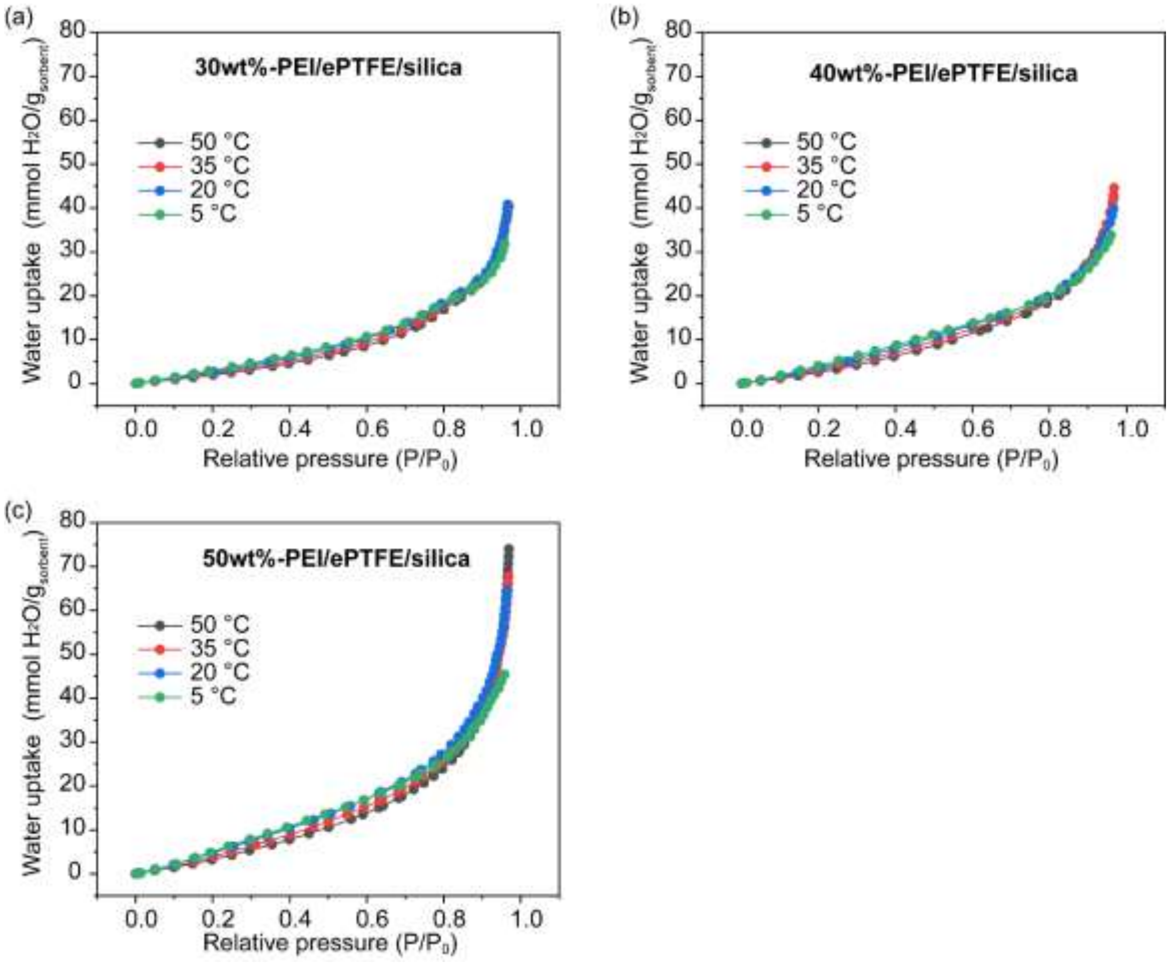

Figure S18. Water adsorption isotherms of (a) 30 wt%, (b) 40 wt%, and (c) 50 wt%-PEI/ePTFE/silica sorbents at 5 °C, 20 °C, 35 °C and 50 °C.

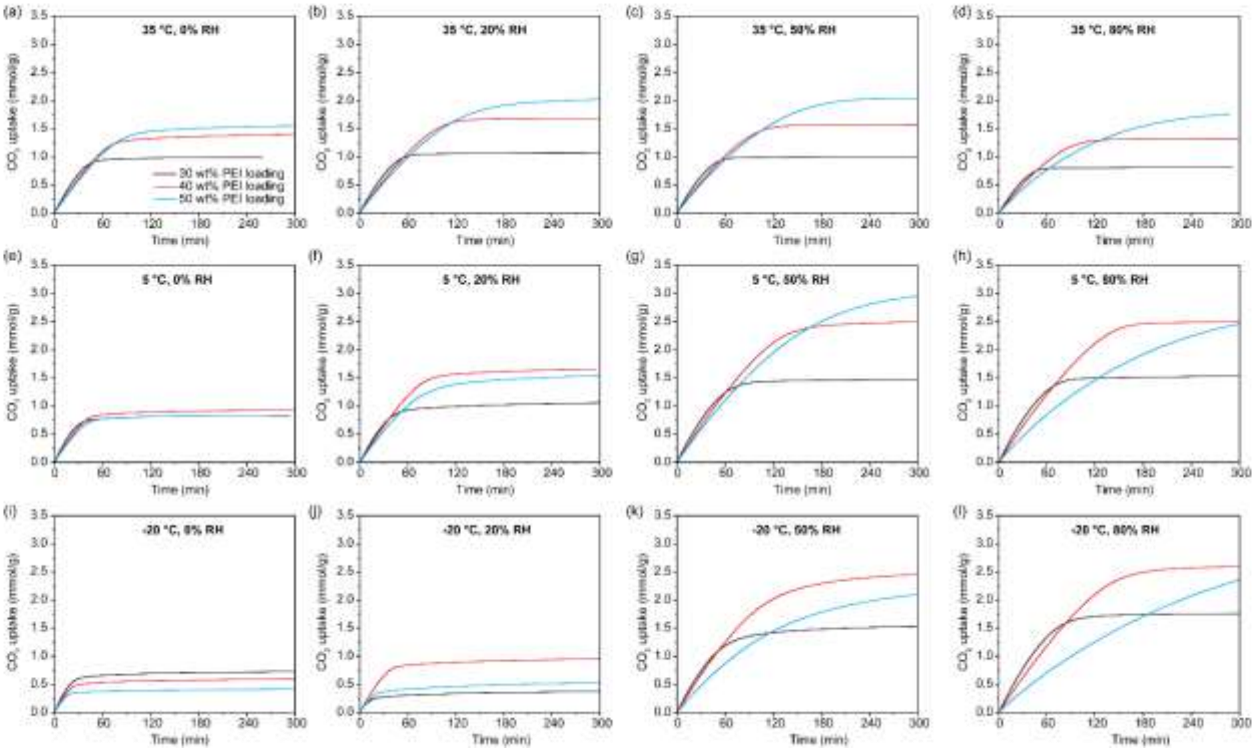

Figure S19. CO_2 uptake profiles (in mmol $CO_2/g_{sorbent}$) of 30 wt%, 40 wt%, and 50 wt%-PEI/ePTFE/silica sorbents under varying temperatures and relative humidities using fixed-bed system. Measurements were conducted under the following conditions: (a) 35 °C, 0% RH, (b) 35 °C, 20% RH, (c) 35 °C, 50% RH, (d) 35 °C, 80% RH, (e) 5 °C, 0% RH, (f) 5 °C, 20% RH, (g) 5 °C, 50% RH, (h) 5 °C, 80% RH, (i) −20 °C, 0% RH, (j) −20 °C, 20% RH, (k) −20 °C, 50% RH and (l) −20 °C, 80% RH.

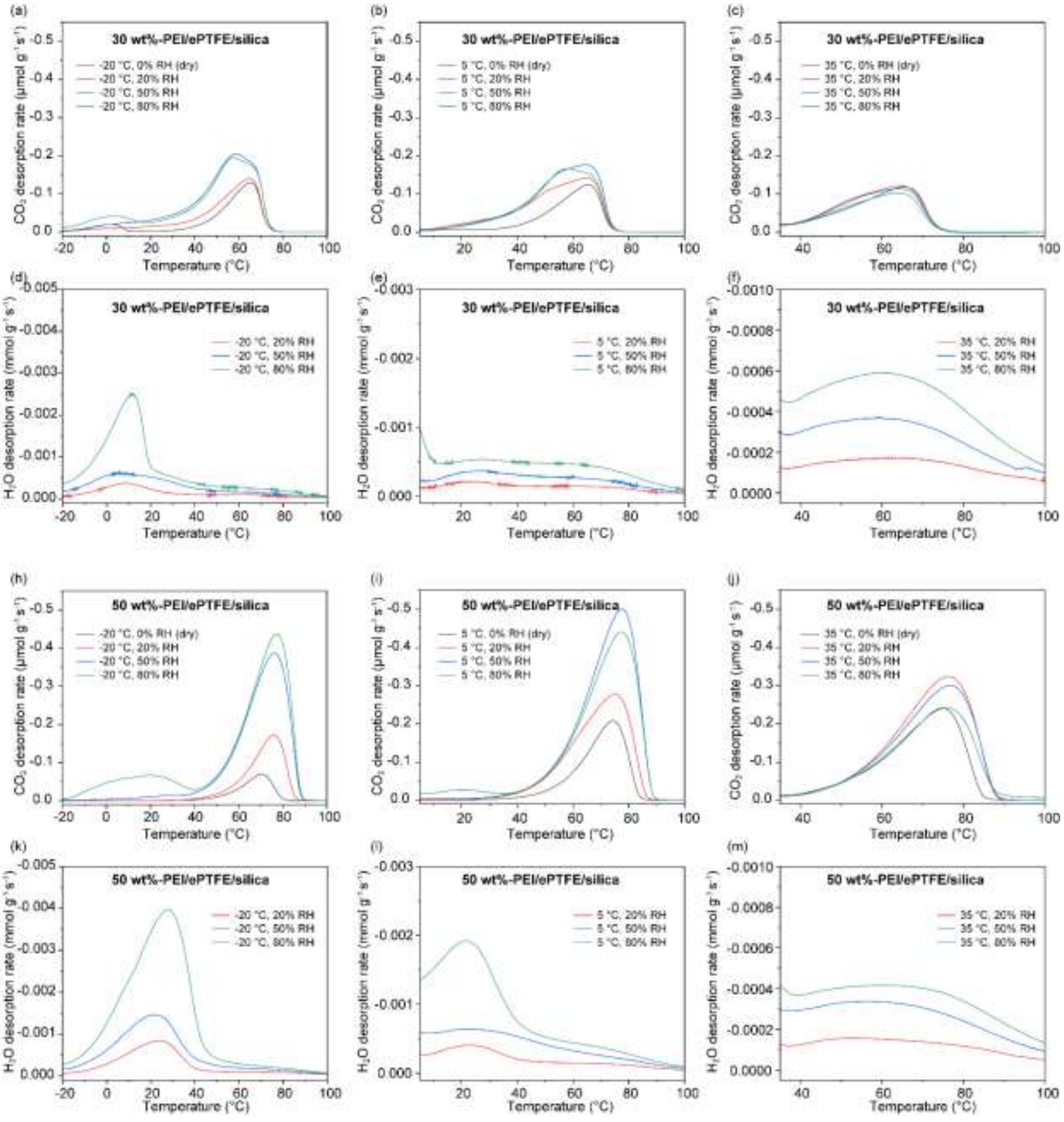

Figure S20. CO_2 TPD profiles of (a–c) 30 wt%-PEI/ePTFE/silica and (h–j) 50 wt%-PEI/ePTFE/silica. H_2O TPD profiles of (d–f) 30 wt%-PEI/ePTFE/silica and (k–m) 50 wt%-PEI/ePTFE/silica. TPD was conducted under N_2 flow (50 mL/min) using a fixed-bed system with the following steps: 2 h pre-purging, temperature ramping at 0.2 °C/min to 100 °C, 1 h isothermal hold at 100 °C, followed by cooling.

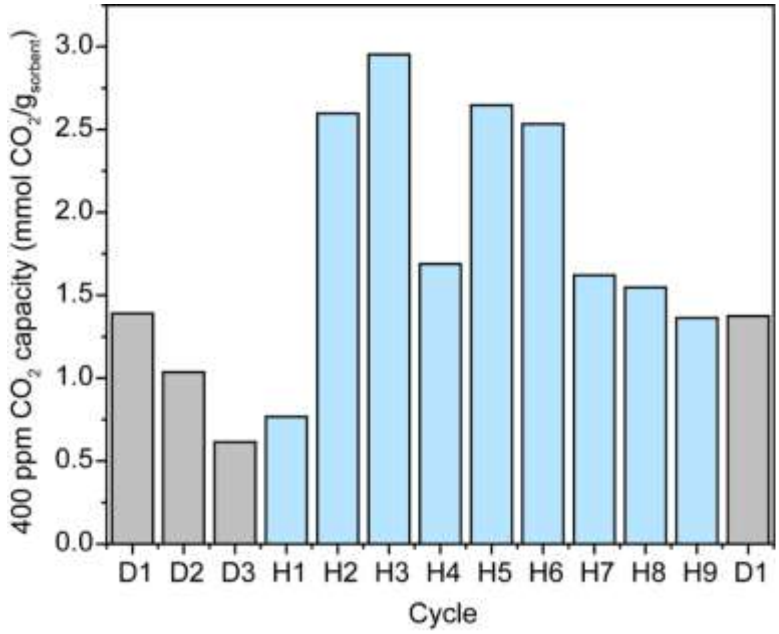

Figure S21. Cyclic CO_2 capacities under dry and humid conditions using 40wt%-PEI/ePTFE/silica. (D1: 35 °C, dry; D2: 5 °C, dry; D3: −20 °C, dry; H1: −20 °C, RH 20%; H2: −20 °C, RH 50%; H3: −20 °C, RH 80%; H4: 5 °C, RH 20%; H5: 5 °C, RH 50%; H6: 5 °C, RH 80%; H7: 35 °C, RH 20%; H8: 35 °C, RH 50%; H9: 35 °C, RH 80%; D1 (repeat): 35 °C, dry).

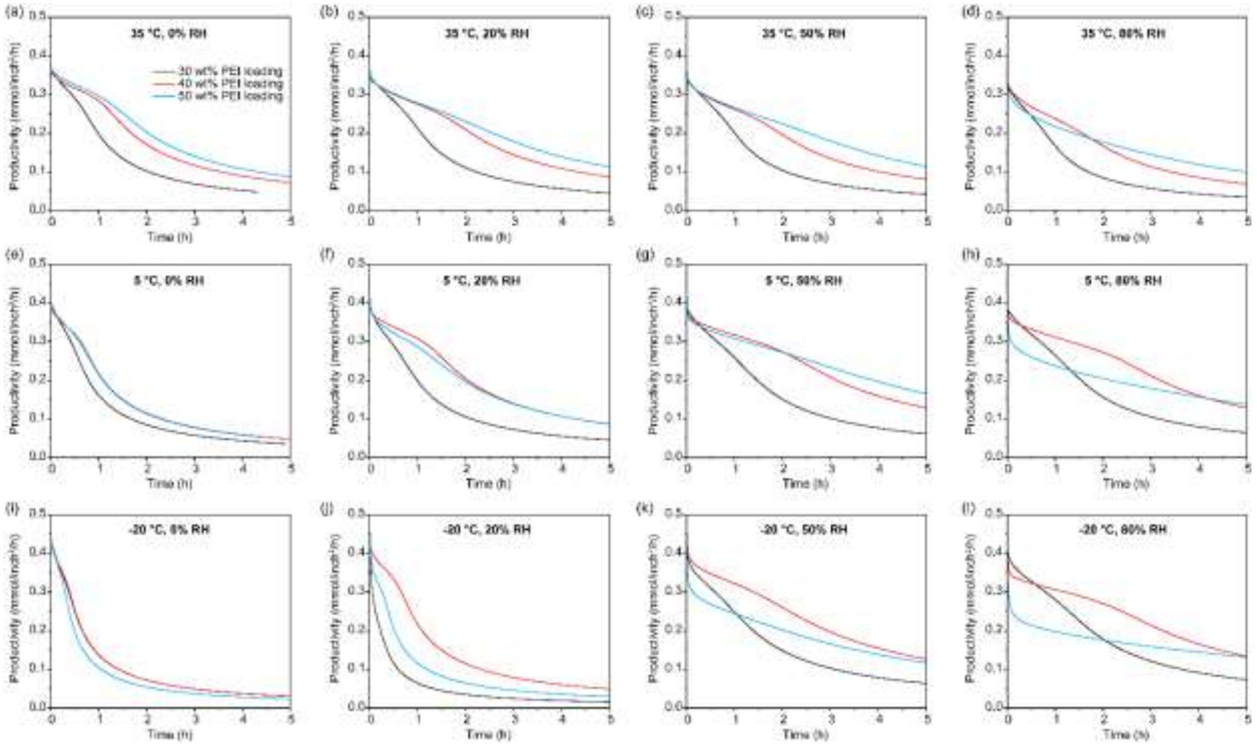

Figure S22. Area-based CO_2 productivity (mmol/inch^2/h) of 30 wt%, 40 wt%, and 50 wt%-PEI/ePTFE/silica sorbents under varying temperatures and relative humidities using fixed-bed system. Measurements were conducted under the following conditions: (a) 35 °C, 0% RH, (b) 35 °C, 20% RH, (c) 35 °C, 50% RH, (d) 35 °C, 80% RH, (e) 5 °C, 0% RH, (f) 5 °C, 20% RH, (g) 5 °C, 50% RH, (h) 5 °C, 80% RH, (i) −20 °C, 0% RH, (j) −20 °C, 20% RH, (k) −20 °C, 50% RH and (l) −20 °C, 80% RH.

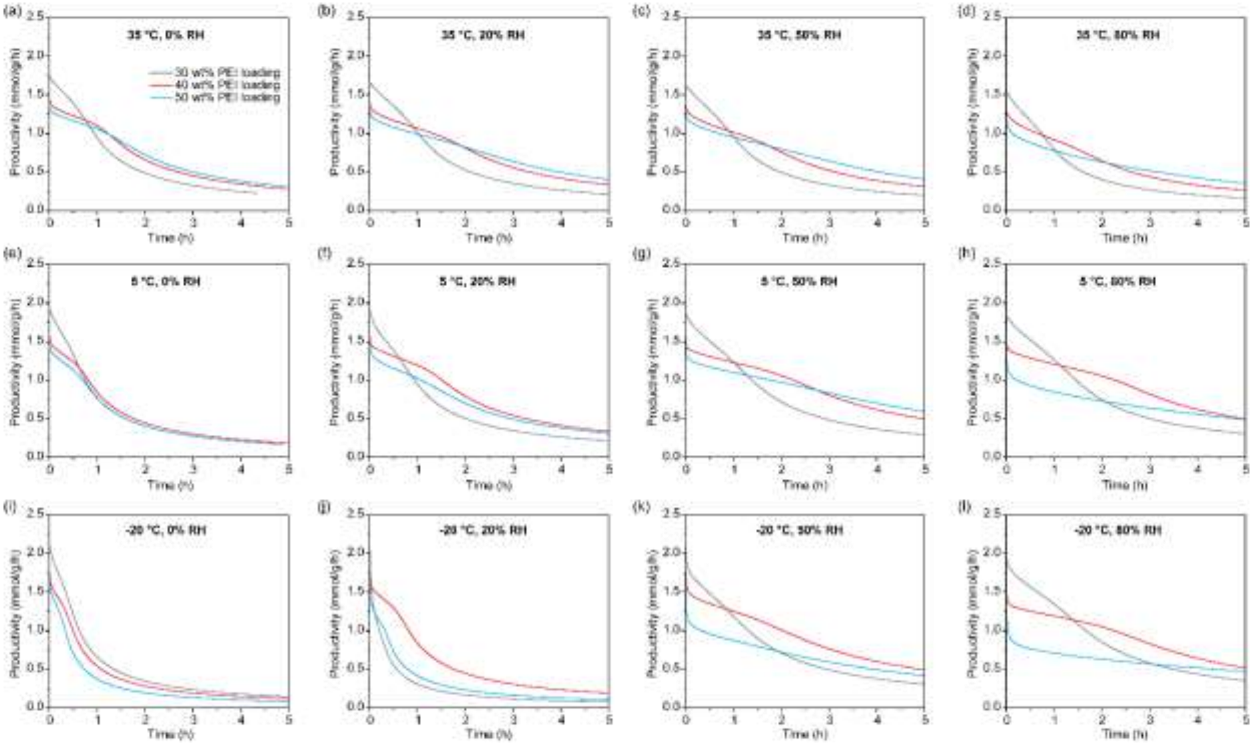

Figure S23. Mass-based CO_2 productivity (mmol/g/h) of 30 wt%, 40 wt%, and 50 wt%-PEI/ePTFE/silica sorbents under varying temperatures and relative humidities using fixed-bed system. Measurements were conducted under the following conditions: (a) 35 °C, 0% RH, (b) 35 °C, 20% RH, (c) 35 °C, 50% RH, (d) 35 °C, 80% RH, (e) 5 °C, 0% RH, (f) 5 °C, 20% RH, (g) 5 °C, 50% RH, (h) 5 °C, 80% RH, (i) −20 °C, 0% RH, (j) −20 °C, 20% RH, (k) −20 °C, 50% RH and (l) −20 °C, 80% RH.

REFERENCES

(1) Greenspan, L. *Humidity Fixed Points of Binary Saturated Aqueous Solutions*; 1977; Vol. 81. https://doi.org/LewisGreenspan.

(2) Min, Y. J.; Ganesan, A.; Realff, M. J.; Jones, C. W. Direct Air Capture of CO_2 Using Poly(Ethyleneimine)-Functionalized Expanded Poly(Tetrafluoroethylene)/Silica Composite Structured Sorbents. *ACS Appl Mater Interfaces* **2022**, *14* (36), 40992–41002. https://doi.org/10.1021/acsami.2c11143.

(3) *Annual Average Relative Humidity by US State*. https://www.currentresults.com/Weather/US/annual-average-humidity-by-state.php (accessed 2025-07-23).

(4) Chrissi Antonopoulos; Theresa L. Gilbride; Evan R. Margiotta; Christian E. Kaltreider. *Guide to Determining Climate Zones by County: Building America and IECC 2021 Updates*; 2022.
